# Oligonucleotide therapies for nonalcoholic steatohepatitis

**DOI:** 10.1016/j.omtn.2024.102184

**Published:** 2024-03-30

**Authors:** Sixu Li, Feng Xiong, Songbo Zhang, Jinghua Liu, Guangping Gao, Jun Xie, Yi Wang

**Affiliations:** 1 Department of Pathophysiology, West China College of Basic Medical Sciences & Forensic Medicine, Sichuan University, Chengdu 610066, China; 2 Department of Cardiology, The Third People’s Hospital of Chengdu, Chengdu 610031, China; 3 Department of Breast Surgery, Sichuan Clinical Research Center for Cancer, Sichuan Cancer Hospital & Institute, Sichuan Cancer Center, Affiliated Cancer Hospital of University of Electronic Science and Technology of China, Chengdu 610041, China; 4 Horae Gene Therapy Center, University of Massachusetts Chan Medical School, Worcester, MA 01605, USA; 5 Department of Microbiology and Physiological Systems, University of Massachusetts Chan Medical School, Worcester, MA 01605, USA; 6 Li Weibo Institute for Rare Diseases Research, University of Massachusetts Chan Medical School, Worcester, MA 01605, USA; 7 Viral Vector Core, University of Massachusetts Chan Medical, School, Worcester, MA 01605, USA

**Keywords:** MT: Oligonucleotides: Therapies and Applications, NASH, NAFLD, RNAi, ASO, siRNA, miRNA, oligonucleotide drug

## Abstract

Nonalcoholic steatohepatitis (NASH) represents a severe disease subtype of nonalcoholic fatty liver disease (NAFLD) that is thought to be highly associated with systemic metabolic abnormalities. It is characterized by a series of substantial liver damage, including hepatocellular steatosis, inflammation, and fibrosis. The end stage of NASH, in some cases, may result in cirrhosis and hepatocellular carcinoma (HCC). Nowadays a large number of investigations are actively under way to test various therapeutic strategies, including emerging oligonucleotide drugs (e.g., antisense oligonucleotide, small interfering RNA, microRNA, mimic/inhibitor RNA, and small activating RNA) that have shown high potential in treating this fatal liver disease. This article systematically reviews the pathogenesis of NASH/NAFLD, the promising druggable targets proven by current studies in chemical compounds or biological drug development, and the feasibility and limitations of oligonucleotide-based therapeutic approaches under clinical or pre-clinical studies.

## Graphical abstract

**Figure:**
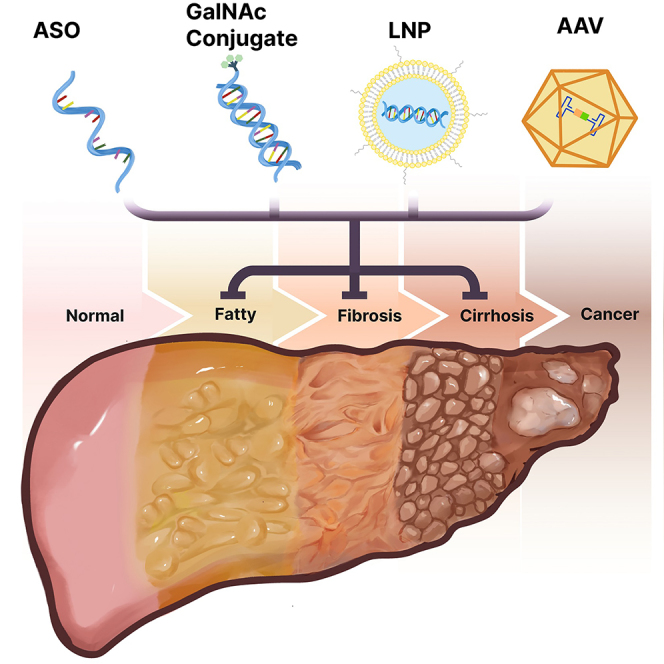

## Teaser

Wang and colleagues summarize pre-clinical and clinical studies that are undertaken by utilizing various types of delivery approaches to testify promising oligonucleotide-related therapeutic strategies for nonalcoholic steatohepatitis.

## Introduction

Nonalcoholic fatty liver disease (NAFLD) (Table 1 for abbreviations) is a clinicopathological syndrome characterized by hepatic steatosis, which lacks secondary causes of excessive fat deposition, such as alcohol.^1^ NAFLD and nonalcoholic steatohepatitis (NASH) have long been considered to be metabolic diseases, as in the majority of NASH patients the disease is also accompanied by metabolic abnormalities, including obesity, insulin resistance (IR) or type 2 diabetes (T2D), hypertriglyceridemia, and dyslipidemia.^2^^,^^3^^,^^4^^,^^5^^,^^6^ Despite obscure symptoms at a very early stage, NASH may gradually progress to cirrhosis and other end-stage liver diseases such as hepatocellular carcinoma (HCC), requiring eventual liver transplantation.^7^ As NASH has brought huge life-threatening concerns and economic burdens around the world, effective therapeutic approaches are urgently desired. It has been widely accepted that NASH results from numerous metabolic and pathologic alterations that proceed in parallel, including genetic predisposition, abnormal lipid metabolism, oxidative stress, lipid toxicity, mitochondrial dysfunction, inflammation, gut dysbiosis, and endoplasmic reticulum (ER) stress,^8^ which certainly raise great difficulties for single-action drug development. So far, the development of chemical drugs targeting thyroid hormone receptor β (Thr-β), glucagon-like peptide 1 receptor (Glp-1R), farnesoid X receptor (Fxr), and peroxisomal proliferator-activated receptor (PPAR) are at the forefront of the drug pipelines.^9^ Followed by the announced positive topline results of Thr-β agonist resmetirom (MGL-3196),^10^ the US Food and Drug Administration (FDA) approved resmetirom as the first-line medication for NASH patients with moderate to advanced liver fibrosis on March 14, 2024,^11^ greatly boosting confidence and demands in NASH-specific drug development.

**Table 1:** List of abbreviations

Abbreviation	Definition
2ʹ-F	2ʹ-fluoro
2ʹ-OMe	2ʹ-*O*-methyl
2ʹ-MOE	2ʹ-*O*-methoxyethyl
AASLD	American Association for the Study of Liver Diseases
AAV	adeno-associated virus
Acc	acetyl-coenzyme A carboxylase
AcNH	*N*-acetylamine
ADA-SCID	adenosine deaminase-deficient severe combined immunodeficiency
ADGRF1	adhesion G-protein-coupled receptor F1
ADR	adverse drug reaction
AdV	adenovirus
AEAA	aminoethyl anisamide
AEG-1	astrocyte elevated gene 1
AGO2	Argonaute 2 protein
AHP	acute hepatic porphyria
ALT	alanine transaminase
AMLN	amylin liver nonalcoholic steatohepatitis
anti-miR	anti-miRNA oligonucleotide
APOC3	apolipoprotein C 3
ApoE	apolipoprotein E
ASGPR	asialoglycoprotein receptor
asiRNA	asymmetric siRNA
ASK1	apoptosis signal-regulating kinase 1
ASO	antisense oligonucleotide
AST	alanine aminotransferase
BDNF	brain-derived neurotrophic factor
BNA	bridged nucleic acid
CAR	chimeric antigen receptor
CCR2/5	C-C chemokine receptor type 2/5
CDAA	choline-deficient/amino acid-defined
CDHFD	choline-deficient high-fat diet
CE	cholesterol esters
CHREBP	carbohydrate response element binding protein
CLCF1	cardiotrophin-like cytokine factor 1
CMV	cytomegalovirus
CpG	cytosine phosphate-guanine
CPP	cell-penetrating peptide
CRN	Clinical Research Network
CYP7A1	cholesterol 7α-hydroxylase
DAMP	damage-associated molecular pattern
DGAT2	diacylglycerol acyltransferase 2
DIO	diet-induced obese
DLinDMA	1, 2-dilinoleyloxy-*N*,*N*-dimethyl-3-aminopropane
DLin-MC3-DMA	dilinoleylmethyl-4-dimethylaminobutyrate
DMD	Duchenne muscular dystrophy
DMN	dimethylnitrosamine
DNL	*de novo* lipogenesis
DSPC	distearolyphosphatidylcholine
dsRNA	double-stranded RNA
EASL	European Association for the Study of the Liver
ECM	extracellular matrix
ENA	ethylene-bridged nucleic acid
ER	endoplasmic reticulum
ESC	enhanced stabilization chemistry
ESC+	enhanced stabilization chemistry-plus
ETC	electron transfer chain
FA	fatty acid
FASN	fatty acid synthase
FDA	US Food and Drug Administration
FFA	free fatty acid
FGF12/19	fibroblast growth factor 12/19
Fxr	farnesoid X receptor
GalNAc	*N*-acetylgalactosamine
GAN	Gubra amylin nonalcoholic steatohepatitis
GI	gastrointestinal
GIP	glucose-dependent insulinotropic polypeptide
GIP-R	insulinotropic polypeptide receptor
GLP-1R	glucagon-like peptide 1 receptor
GLU-R	glucagon receptors
GNA	glycol nucleic acid
GPCR	G-protein-coupled receptor
GWAS	genome-wide association study
HAO1	hydroxyacid oxidase 1
hATTR	hereditary transthyretin
HBV	hepatitis B virus
HCC	hepatocellular carcinoma
HCV	hepatitis C virus
HF/HS	high fat and sucrose
HFD	high-fat diet
HFFC	high-fat/fructose/cholesterol
HFHCD	high-fat/cholesterol diet
HMGB1	high-mobility group box 1
HSC	hepatic stellate cell
HSD17B13	17β-hydroxysteroid dehydrogenase 13
HSP47	47-kDa heat-shock protein
HULC	highly upregulated in liver cancer
ICAM-1	intercellular adhesion molecule 1
ICC	interstitial cell of Cajal
Ihh	Indian hedgehog
IPF	idiopathic pulmonary fibrosis
IR	insulin resistance
JNK	c-Jun N-terminal kinase
KC	Kupffer cell
KLF11	Krüppel-like factor 11
LD	lipid droplet
LDLR	low-density lipoprotein receptor
LICA	ligand-conjugated antisense oligonucleotide
LNA	locked nucleic acid
lncRNA	long non-coding RNA
LNP	lipid nanoparticles
LPH	lipid-protamine-hyaluronic acid
LV	lentivirus
LXR	liver X receptor
MALAT1	metastasis-associated lung adenocarcinoma transcript 1
MAP	mitogen-activated protein
MAPK	mitogen-activated protein kinase
MAPKKK	mitogen-activated protein kinase kinase kinase
MASH	metabolic dysfunction-associated steatohepatitis
MASLD	metabolic dysfunction-associated steatotic liver disease
MCD	deficient in methionine and choline
MCJ	methylation-controlled J protein
miRNA	microRNA
MoMF	monocyte-derived macrophages
mRNA	messenger RNA
MST3	mammalian sterile 20-like 3
mtDNA	mitochondrial DNA
NAFLD	nonalcoholic fatty liver disease
NAS	nonalcoholic fatty liver disease activity scoring
NASH	nonalcoholic steatohepatitis
ncRNA	non-coding RNA
NDA	new drug application
NEAT1	nuclear paraspeckle assembly transcript 1
NF-кB	nuclear factor κB
OCA	obeticholic acid
Opn	osteopontin
PAMAM	poly-amidoamine
PANK	pantothenate kinase
PARP	potential poly(adenosine 5′-diphosphate ribose) polymerase
PCSK7	proprotein convertase subtilisin/kexin type 7
PEG	polyethylene glycol
PGC-1α	peroxisomal proliferator-activated receptor γ co-activator 1α
PH1	primary hyperoxaluria type 1
PKLR	pyruvate kinase L/R
PMO	phosphorodiamidate morpholino oligomers
PNA	peptide nucleic acid
PNPLA3	patatin-like phospholipase domain-containing 3
PO	phosphodiester
PPAR	peroxisomal proliferator-activated receptor
pri-miRNA	primary miRNA
PRR	pattern-recognition receptor
PS	phosphorothioate
RISC	RNA-induced silencing complex
RNAi	RNA interference
ROS	reactive oxygen species
SAHA	suberanilohydroxamic acid
SalB	salvianolic acid B
saRNA	small activating RNA
Scd	stearoyl-CoA dehydrogenase
SGLT-1/2	sodium-glucose co-transporter 1/2
shRNA	short hairpin RNA
siRNA	short interfering RNA
SIRT1	silent information regulator 1
SMS1	sphingomyelin synthase 1
SREBF2	sterol-regulatory element binding factor 2
SREBP1c	sterol-regulatory element binding protein 1c
STC	standard template chemistry
STK	serine/threonine protein kinase
T2D	type 2 diabetes
T3	tri-iodothyronine
tcDNA	tricyclo-DNA
TG	triglyceride
THR-β	thyroid hormone receptor β
TLR	Toll-like receptor
TNF-α	tumor necrosis factor
TRBP	transactivation-responsive RNA-binding protein
TTR	transthyretin
TZD	thiazolidinedione
UNA	unlocked nucleic acid
UPR	unfolded protein response
UTR	untranslated region
VAP-1	vascular adhesion protein-1
VLDL	very-low-density lipoproteins
WAT	white adipose tissue
YAP	Yes-associated protein

As an emerging drug-development strategy, oligonucleotide drugs have risen rapidly in recent years. Oligonucleotides refer to small DNA/RNA molecules with 8–50 nucleotides in length that bind to target RNA via Watson-Crick base pairing.^12^ Oligonucleotides can be normally used to inhibit gene expression through various mechanisms including RNA interference (RNAi), RNase H-mediated cleavage, and non-coding RNA (ncRNA) inhibition.^13^ Thanks to their potent gene-silencing capacity, oligonucleotides have been widely applied in gene therapy via both vehicle-based and vehicle-free approaches.^13^^,^^14^ Liver is considered an attractive organ for gene therapy due to natural hepatic tropism for many virus or non-viral vehicles.^15^^,^^16^ Therefore, oligonucleotide drugs are now rendering potential therapeutic options for patients with various metabolic liver diseases.^15^ For instance, givosiran and mipormersen are two oligonucleotide drugs approved for treating acute hepatic porphyria (AHP)^17^ and homozygous familial hypercholesterolemia (HoFH),^18^ which have encouraged attempts to develop oligonucleotides in the treatment of NASH. Here, we summarize the latest advances and perspectives in NASH/NAFLD pathogenesis, chemical compounds undergoing NASH-related clinical investigations, and recent innovations in liver-targeting therapeutic oligonucleotides for NAFLD/NASH.

## Clinical presentation and diagnosis

In 1980 NASH was, for the first time, described as a nonalcoholic disease with similar pathological features and tendency to cirrhosis as alcoholic hepatitis.^19^ The consensus statement published in 2022 reported that the global prevalence of NAFLD in adults was estimated to range from 23% to 25%, among whom 1 in 5 were diagnosed with NASH.^20^ In the United States, the number of people with NASH was predicted to reach 19.53 million by 2039.^21^ Nowadays, NAFLD/NASH is considered to be greatly driven by altered metabolism, whereby many metabolic factors are involved.^22^ To further strengthen the consensus from the field, in June 2023, the European Association for the Study of the Liver (EASL) Congress announced the new nomenclatures MASH (metabolic dysfunction-associated steatohepatitis) and MASLD (metabolic dysfunction-associated steatotic liver disease) to replace NASH and NAFLD, respectively.^23^ Based on 14 histological features assigned in the NAFLD activity scoring (NAS) system that was designed by the Pathology Committee of the NASH Clinical Research Network (CRN), scores reaching 5 or above correlate with increased severities of NASH diagnosis.^24^ Additionally, according to the practice guidance from the American Association for the Study of Liver Diseases (AASLD), patients presenting more than 5% hepatocyte steatosis and lobular inflammation (regardless of liver fibrosis) but lacking excessive alcohol consumption are diagnosed with NASH.^1^ Noninvasive assessments (including “NAFLD fibrosis scoring” or “fibrosis-4 scoring,” magnetic resonance elastography, ultrasound elastography, and vibration-controlled transient elastography) are usually needed for patients with comorbid conditions, persistently elevated transaminases, and/or concern for cirrhosis.^25^ Liver biopsy, as the only method to distinguish simple liver fatty infiltration from NASH, should be considered once inconclusive results of fibrosis are obtained from the aforementioned diagnostic methods.^25^ However, patients typically do not undergo liver function tests or imaging diagnosis until symptoms occur, resulting in progressive NASH conditions ahead of the time of discovery. Therefore, NASH is also known as the “silent killer.”

## Pathogenesis and current treatment approaches for NASH

NASH differs from simple steatosis by showing more significant hepatocyte apoptosis accompanied by increased inflammation.^26^ The “two-hit hypothesis” suggested that NASH development requires steatosis caused by triglyceride (TG) accumulation and oxidative stress-mediated lipid peroxidation.^27^ Further studies held the “non-triglyceride lipotoxicity hypothesis” by elucidating that TGs played protective roles throughout NASH progression, whereas liver injury was mainly caused by non-triglyceride lipotoxic metabolites.^28^ In many cases, liver inflammation prior to steatosis was observed, leading to the prevailing “multiple parallel hits hypothesis” that NASH is the result of multiple factors derived especially from adipose tissue and gut.^29^ Overload of fatty acids (FAs) in the liver has been shown to contribute to IR and lipotoxicity^30^^,^^31^^,^^32^ via disrupted mitochondria respiration^33^^,^^34^ and elevated reactive oxygen species (ROS) to cause hepatocyte death.^35^^,^^36^ The aforementioned cellular stress could stimulate pro-inflammatory and pro-fibrogenic responses of immune cells including monocyte-derived macrophages, resident Kupffer cells (KCs), and lymphocytes,^37^^,^^38^ which in turn promote extracellular matrix (ECM) production and fibrosis via activated hepatic stellate cells (HSCs).^39^ Moreover, toxic bile acid retention caused by disturbed hepatobiliary function has been found to be involved in NASH pathogenesis.^40^^,^^41^ Furthermore, recent studies have demonstrated the roles of bacterial metabolites and increased gut permeability in the progression of NAFLD/NASH.^42^^,^^43^

Nowadays, primary treatments of NAFLD still mainly focus on lifestyle intervention. For example, limiting fructose intake is thought to improve disease conditions, as daily fructose ingestion has been shown to associate with liver fibrosis in NAFLD patients.^44^ In addition, aerobic exercise and adequate sleep are beneficial.^45^^,^^46^ Nevertheless, the efficacies of such interventions mainly rely on individuals’ genetic backgrounds and/or self-discipline. Once the disease has progressed to fibrotic stages, lifestyle interventions are considered meaningless. Prior to the recent approval of resmetirom by the FDA, medications for NASH mainly aim at harnessing risk factors, including correcting dyslipidemia and hyperglycemia. For instance, vitamin E has been used in the treatment of NASH for its antioxidant properties.^47^ Some thiazolidinediones (TZDs), such as pioglitazone, have been shown to act as insulin sensitizers to improve metabolic status.^48^^,^^49^ However, the data also indicated the increased number of adverse events in pioglitazone-administered NASH patients by showing weight gain, dysregulated bone metabolism, and hemorrhagic stroke.^48^^,^^49^

## Current chemical drug-developing strategies for NASH

### Targeting lipid metabolism

**Figure 1:**
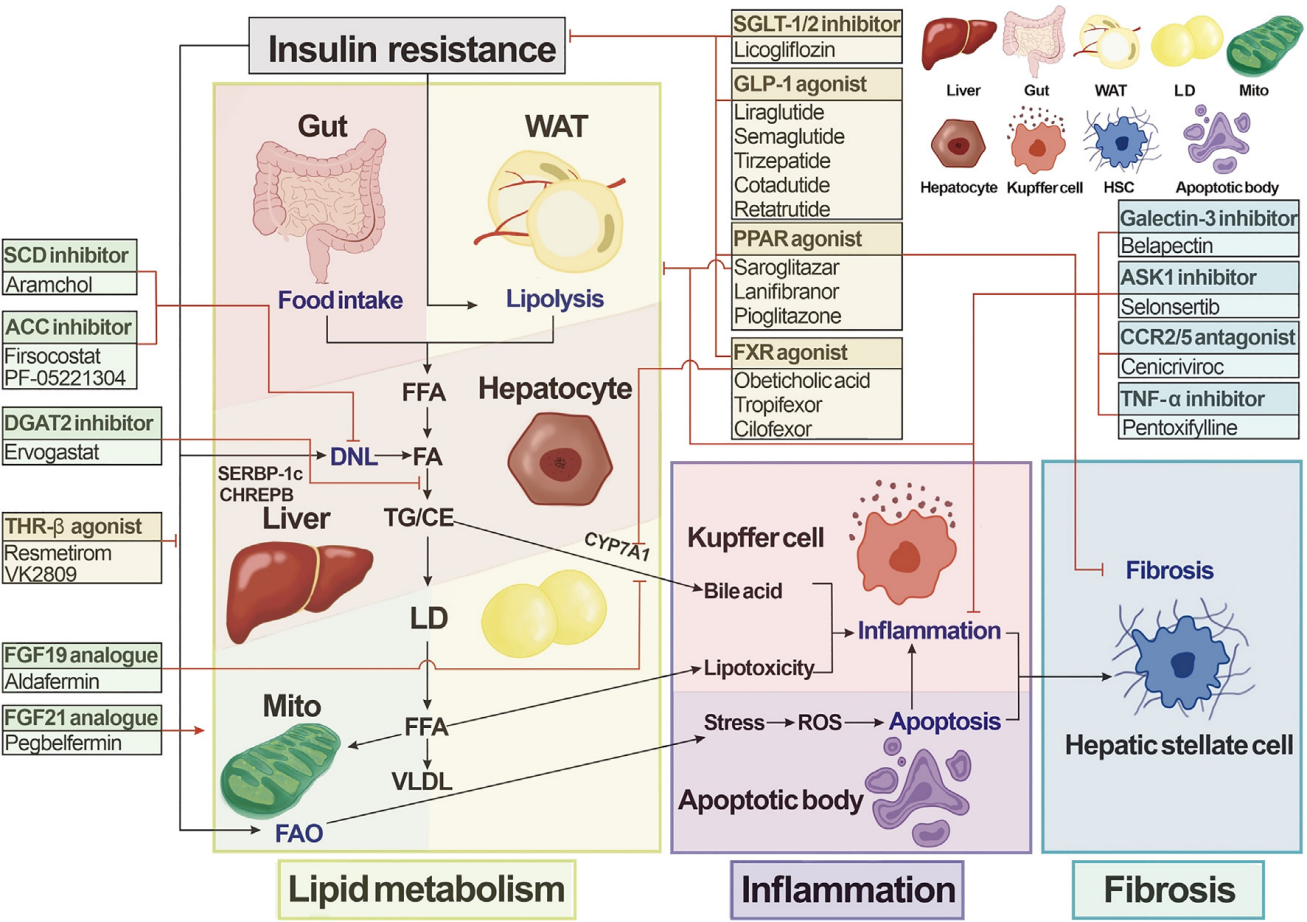
Small-molecule drugs and biologics for NASH therapy Several small-molecule drugs and biologics for nonalcoholic steatohepatitis (NASH) are now in development, including the projects now closed. Drugs are categorized according to their targets in the NASH pathogenesis. SCD, stearoyl-CoA dehydrogenase; ACC, acetyl-CoA carboxylase; DGAT2, diacylglycerol acyltransferase 2; SGLT-1/2, sodium-glucose co-transporter 1/2; GLP, glucagon-like peptide-1 receptor; PPAR, peroxisomal proliferator-activated receptor; THR-β, thyroid hormone receptor β; FXR, farnesoid X receptor; ASK1, apoptosis signal-regulating kinase 1; CCR2/5, C-C chemokine receptor type 2/5; FGF 19/21, fibroblast growth factor 19/21; WAT, white adipose tissue; FFA, free fatty acid; DNL, *de novo* lipogenesis; TG, triglyceride; CE, cholesteryl ester; SREBP-1c, sterol-regulatory element binding protein 1c; CHREBP, carbohydrate response element binding protein; CYP7A1, cholesterol 7α-hydroxylase; LD, lipid droplet; VLDL, very-low-density lipoproteins; FAO, fatty acid oxidation; mito, mitochondria; ROS, reactive oxygen species; HSC, hepatic stellate cell.

Dietary fat intake, plasma free fatty acid (FFA) absorption, and *de novo* lipogenesis (DNL) provide major sources for hepatic lipids. Once esterified to TGs and cholesterol esters (CEs), the excessive FAs are stored in lipid droplets (LDs), where fatty acid oxidation (FAO) and very-low-density lipoprotein (VLDL) secretion are important outlets for them^50^ (Figure 1). Any faulty step can render opportunities to develop liver steatosis.^51^^,^^52^ For instance, enhanced DNL promoted liver fat accumulation,^53^ while significantly elevated FFA levels were also shown in NAFLD patients.^54^ In addition, the enzymatic activity of β-hydroxyacyl-coenzyme A (CoA) dehydrogenase (the rate-limiting enzyme for β-oxidation in FAO) was shown to decrease during the progression of NAFLD/NASH.^55^ Therefore, key enzymes involved in this pathway (including stearoyl-CoA dehydrogenase [Scd], acetyl-CoA carboxylase [Acc], fatty acid synthase [Fasn], diacylglycerol acyltransferase 2 [Dgat2], and fibroblast growth factor 21 [Fgf21] and Fgf19) for generating liver FAs/TGs have been shown to serve as potential therapeutic targets to prevent NASH progression.

Based on this knowledge, Acc and Scd inhibitors were reported to ameliorate NASH by reducing steatosis, liver injury, inflammation, and fibrosis.^56^^,^^57^ A phase 2 study of liver-targeted Acc inhibitor firsocostat (GS-0976) has been completed (NCT02856555), showing decreased hepatic steatosis and fibrosis compared with placebo groups.^58^^,^^59^ Another Acc inhibitor, clesacostat (PF-05221304), was shown to possess an anti-steatosis effect in high-dose groups (NCT03248882).^60^ Meanwhile, the Scd inhibitor aramchol was reported to significantly alleviate liver fibrosis in NASH patients^61^ and is now awaiting the phase 3 study for formulation improvement (NCT04104321).^62^ Fasn is another enzyme in the DNL pathway, and the misregulated expression of this factor was found to mediate pro-inflammatory and fibrogenic signaling.^63^ Recently, TVB-2640 (denifanstat), a Fasn inhibitor, finished its phase 2 trial in NASH patients (NCT04906421). On the other hand, liver FAs can also be used for TG synthesis via Dgat2 catalysis^64^ (Figure 1). The phase 2 study of the Dgat2 selective inhibitor ervogastat (PF-06865571) co-administered with clesacostat in NAFLD patients has been completed with satisfactory results (NCT03776175).^60^ Fgf 21 and Fgf19 could serve as diagnostic markers for NASH.^65^ Fgf19 also regulates cholesterol 7α-hydroxylase (*CYP7A1*) gene transcription, which encodes the rate-limiting enzyme in bile acid synthesis.^66^ Fgf21/19 and their analogs were shown to reduce hepatic steatosis, inflammation, and fibrosis in NASH mouse models.^67^^,^^68^^,^^69^ Currently, Fgf21 analogs pegbelfermin (BMS-986036) and Fgf19 analog aldafermin (NGM282) were found to present significant therapeutic effects in NASH patients (NCT03486899 and NCT03912532).^70^^,^^71^^,^^72^^,^^73^^,^^74^^,^^75^

### Targeting insulin resistance

IR results in higher insulin levels than normal because insulin-targeted tissues are less responsive in blood sugar regulation.^76^ It has been widely accepted that IR is involved in the progression of liver steatosis and fibrosis.^77^^,^^78^^,^^79^ IR-mediated lipid metabolism disturbance may contribute to NAFLD/NASH through promotion of white adipose tissue (WAT) lipolysis and liver DNL as well as altered mitochondrial FAO.^80^ For example, in NAFLD patients, serum FFA levels increased due to the failure of insulin-mediated lipolysis suppression.^78^ Meanwhile, hyperinsulinemia and hyperglycemia in NASH patients may activate sterol-regulatory element binding protein 1c (Srebp1c) and carbohydrate response element binding protein (Chrebp), respectively, to activate DNL-related gene expression in the liver.^81^ Mitochondrial FA β-oxidation may increase to adapt to the upregulated lipogenesis at an earlier stage, but decompensates to such changes, eventually leading to mitochondria damage, oxidative stress, and insulin signaling impairment.^80^ Currently, promising therapeutic targets involved in the clinical treatments of IR-related NASH include Glp-1R, Thr-β, sodium-glucose co-transporter 1/2 (Sglt1/2), PPAR, and Fxr.

As glucose-lowering drugs, Glp-1R agonists have been approved for treating T2D,^82^ and were also shown to protect lipid metabolism homeostasis and improve liver function.^83^^,^^84^^,^^85^^,^^86^ An FDA-approved long-acting Glp-1 analog, liraglutide, was shown in a phase 2 study (NCT01237119) to improve liver function and resolve pathological manifestations in NASH individuals with or without T2D.^87^^,^^88^ During single administration or in combinatory treatment with cilofexor or firsocostat, semaglutide has been shown to resolve hepatocyte inflammation and ballooning, alleviate liver steatosis, or even impede liver fibrosis in phase 2 studies (NCT02970942, NCT03987451, NCT03987074, and NCT04971785).^89^^,^^90^^,^^91^^,^^92^ A phase 3 research study of single administration of semaglutide in NASH is under way (NCT04822181). However, semaglutide and liraglutide were unfortunately shown to be associated with increased risk of gastrointestinal adverse events in weight control.^93^ Additionally, serving as dual agonists for both Glp-1Rs and glucose-dependent insulinotropic polypeptide (Gip) receptors (Gip-Rs), tirzepatide (LY3298176) and cotadutide (MEDI0382) are also undergoing clinical trials of NASH therapy (NCT04166773 and NCT04019561). Furthermore, efinopegdutide (MK-6024), the dual agonist for Glp-1Rs and glucagon receptors (Glu-Rs), has been granted a fast-track designation from the FDA recently for NASH treatment (NCT04944992). Retatrutide (LY3437943) is a triagonist of Glp-1Rs, Gip-Rs, and Glu-Rs. In recently published phase 2 results, retatrutide was demonstrated to resolve hepatic steatosis in obese patients with NASH (NCT04881760).^94^ The Thr-β ligand tri-iodothyronine (T3) has been shown to confer insulin-like effects by regulating functional gene expression in FA synthesis.^95^ The positive topline results of the Thr-β-selective agonist resmetirom (MGL-3196) in the phase 3 trial (NCT03900429) were announced in December 2023.^10^ Very recently, it has been approved by the FDA as the first NASH-specific drug for treating patients with moderate to advanced liver fibrosis.^11^ The phase 2 study of another Thr-β agonist, VK2809, is under way to treat histologically confirmed NASH patients (NCT04173065). Sglt1 and Sglt2 are glucose transporters that mediate uptake through the apical cell membrane.^96^ Sglt1 is mainly responsible for sodium-dependent glucose uptake in the small intestine, while Sglt2 is responsible for glucose reabsorption in renal proximal convoluted tubules.^97^^,^^98^ Licogliflozin (LIK066), a chemical compound inhibiting both Sglt1 and Sglt2, was found to improve the liver function in obese patients with NASH in a phase 2 study (NCT03205150).^99^ The PPAR family members PPAR-α, PPAR-β/δ, and PPAR-γ have also been demonstrated to link with NASH via regulating lipogenesis,^100^^,^^101^ FA transportation,^102^ and energy utilization,^103^^,^^104^^,^^105^ as well as lipotoxicity-related inflammation.^106^ Saroglitazar has been shown to act as a PPAR-α/γ agonist, decreasing liver fat content and alanine transaminase (ALT) in NAFLD/NASH patients (NCT03061721).^107^ Lanifibranor (IVA337), a pan-PPAR ligand that stimulates PPAR-α, -δ, and -γ, was reported to decrease the SAF (steatosis, activity, and fibrosis) score in patients with active NASH^108^^,^^109^ and is now in a phase 3 study (NCT04849728). However, the PPAR-γ-specific agonist pioglitazone was recently shown to have no increased benefit over placebo in NASH patients without diabetes (NCT00063622).^110^ The phase 3 study of elafibranor, which activates PPAR-α and PPAR-δ, was also terminated due to low efficacy (NCT02704403).^111^^,^^112^ It has been shown that the bile acid receptor Fxr downregulates Cyp7a1 expression to lower bile acid level.^40^^,^^113^^,^^114^ Fxr activation was also found to inhibit the expression of Srebp1c and facilitate TG homeostasis.^115^ The phase 3 study (NCT02548351) of obeticholic acid (OCA), an Fxr agonist that was shown to decrease IR in NAFLD patients,^116^ is now terminated. Tropifexor (LJN452) has been shown to downregulate alanine aminotransferase (AST) level and hepatic fat fraction in NASH patients,^117^ but its phase 2 study was terminated (NCT02855164). Cilofexor (GS-9674), another Fxr agonist, is now in a combination therapy study with tropifexor (NCT03449446).^118^

### Targeting hepatocyte inflammation, fibrosis, and death

As mentioned earlier, increased serum FFAs and accumulated lipids in the liver could both cause liver steatosis, where lipotoxicity is considered one of the most critical mechanisms leading to the transition of NASH from NAFLD.^119^ Under such circumstances, hepatocyte apoptosis is induced by subsequent oxidative stress, ER stress, and other damage,^120^^,^^121^^,^^122^ which in turn cause inflammation and fibrosis via activated KCs and HSCs, respectively.^123^^,^^124^ Hence, anti-inflammation/anti-fibrosis strategies for treating NASH are considered effective by manipulating the targets including C-C chemokine receptor type 2/5 (Ccr2/5), tumor necrosis factor (TNF-α), vascular adhesion protein 1 (Vap-1), galectin-3, and apoptosis signal-regulating kinase 1 (Ask1).

It has been shown that Ccr2-mediated hepatic infiltration of monocyte-derived macrophages (MoMFs) could directly cause inflammation and activate HSCs.^125^ Ccr5, another member of the Ccr family expressed on HSCs, has also been shown to promote HSC migration, proliferation, and secretion.^126^^,^^127^ Cenicriviroc (CVC), a dual inhibitor of Ccr2/5, had its phase 3 clinical trials in treating NASH terminated early due to lack of efficacy (NCT03028740).^128^ Pentoxifylline (PTX), a methylxanthine derivative attenuating the production of pro-inflammatory cytokines including TNF-α,^129^ was shown to improve the histological features of NASH (NCT00590161) and is now in a phase 3 study (NCT05284448).^130^ Vap-1, also known as semicarbazide-sensitive amine oxidase, promotes the recruitment of pro-inflammatory cells to the liver.^131^ The phase 1 clinical trial of its inhibitor TERN-201 has been completed (NCT04897594). Moreover, galectin-3 is a glycan-binding protein that has been shown to activate HSCs or myofibroblasts, which contributes to tissue fibrogenesis.^132^^,^^133^^,^^134^^,^^135^^,^^136^ The galectin-3 inhibitor belapectin (GR-MD-02) was reported to reduce liver fibrosis in NASH patients in a phase 2 study (NCT02421094). Selonsertib (GS-4997) is a selective inhibitor targeting Ask1, a mitogen-activated protein (Map) kinase kinase kinase (Mapkkk), in response to various cytotoxic stresses.^137^ The therapeutic potential of selonsertib was shown in combination with firsocostat or cilofexor in a phase 2 study for treating bridging fibrosis or compensated cirrhosis due to NASH (NCT03449446 and NCT02781584).^138^ Notably, ER stress initiated by failed unfolded protein response (UPR) network is proven to be associated not only with metabolism disorders but also with inflammation and apoptosis.^120^ The AdipoR1/AdipoR2 dual agonist peptide JT003 was shown to regulate ER functions and improve liver fibrosis in mouse models.^139^ Another recent study has demonstrated that BGP-15, a potential poly (adenosine 5′-diphosphate ribose) polymerase (PARP) inhibitor, functioned in ER stress blockade and NASH mitigation when combined with olamkicept (sgp130Fc, an interleukin-6 *trans*-signaling blocker).^140^^,^^141^

## Oligonucleotide drug-development strategies for liver diseases

Although major obstacles including relatively lower therapeutic efficacy and tissue specificity compared with conventional chemical compounds prevent the widespread application of oligonucleotide drugs, as of December 2023 dozens of oligonucleotide drugs have received regulatory approval from the FDA. Given the high perfusion rate, discontinuous sinusoidal endothelium, and abundant receptors in the liver, oligonucleotide drugs have long been considered as the alternative approach to treat liver metabolic diseases.^142^ Among these approved drugs, 11 target the liver. Intensive studies in oligonucleotide therapies have shed light on treating various liver diseases, including NASH. Learning from valuable results obtained in NASH-related chemical compounds and biologics development (Figure 1), oligonucleotide drugs have been designed to target critical factors residing in, but not limited to, the aforementioned pathways.

### Type of oligonucleotides and the modes of action

As small synthetic nucleic acid polymers, oligonucleotides target messenger RNA (mRNA), ncRNA, or DNA via complementary base pairing while also interacting with certain proteins through three-dimensional binding.^143^ Currently, antisense oligonucleotide (ASO), small interfering RNA (siRNA), microRNA (miRNA) mimic or inhibitor, and small activating RNA (saRNA) are the most intensively studied oligonucleotide species, with diversified action modes, including expression inhibition or activation of functional genes and non-coding transcripts as well as mRNA splicing modulation.^144^

#### ASO

**Figure 2:**
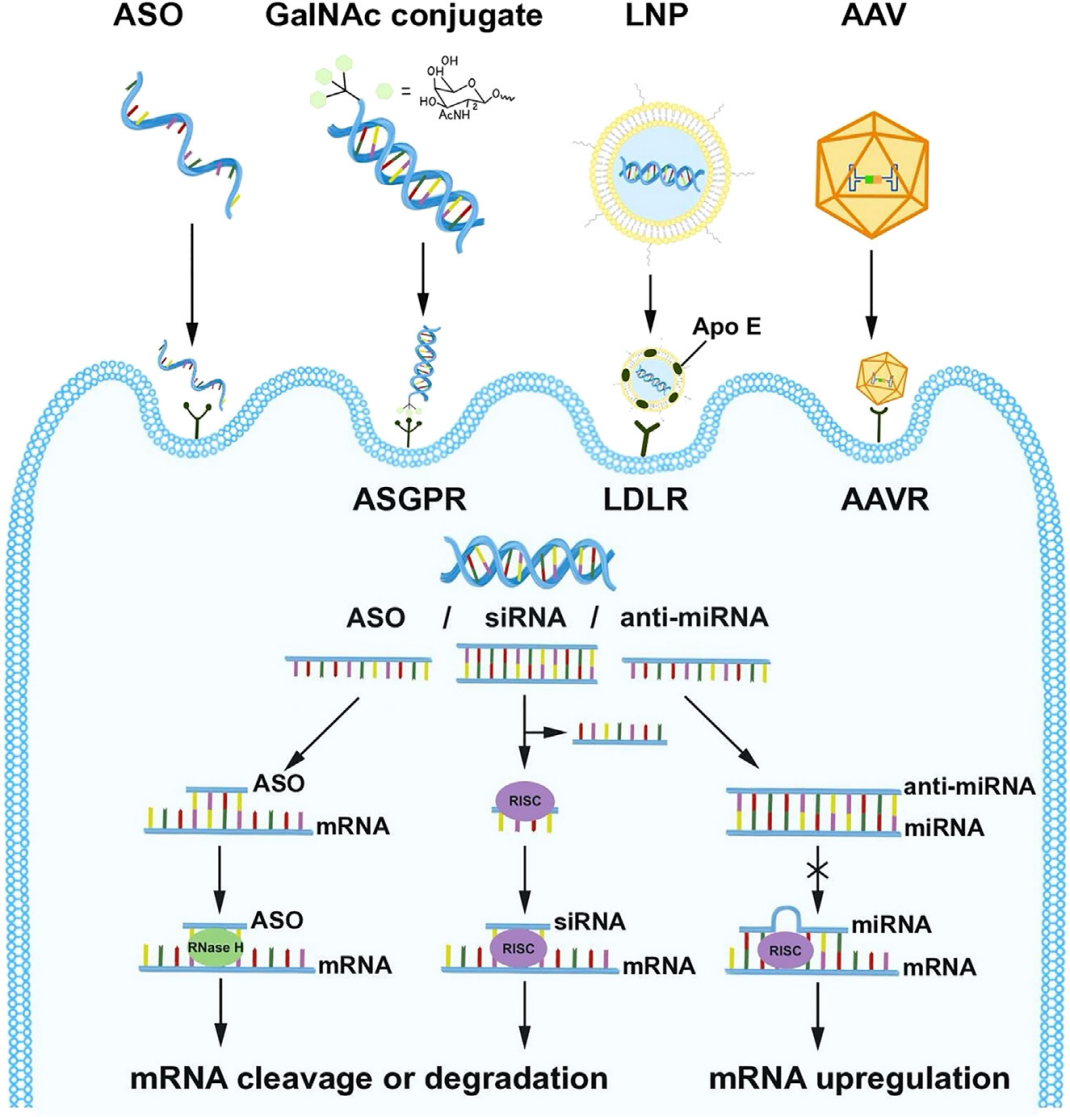
Hepatic delivery systems and action modes of oligonucleotides for liver diseases GalNAc-ASO/siRNA conjugates are delivered to hepatocytes via the ASGPR expressed on hepatocyte surface. LNPs containing siRNA/miRNA are internalized via the LDLR expressed on the hepatocyte surface. AAV delivers shRNA and miRNA for gene knockdown via primary cell-surface glycoprotein receptors and secondary receptors or universal AAV receptor (AAVR). ASO binds to the target mRNA and attracts RNase H for mRNA degradation. siRNA is loaded into the RISC, leading to targeted mRNA cleavage or translation inhibition. miRNA complementary base pairs with the corresponding miRNA to block the cleavage of targeted mRNA via RISC. GalNAc, N-acetylgalactosamine; LNP, lipid nanoparticle; AAV. adeno-associated virus; LDLR, low-density lipoprotein receptor.

ASO is defined as a short, synthetic, single-stranded DNA, consisting of 8–50 nucleotides in length and designed to bind to RNA via Watson-Crick base pairing.^145^^,^^146^ Currently, ASOs make up more than 60% of oligonucleotide drugs undergoing active development.^144^ Fomivirsen is the first FDA-approved ASO drug developed for treating cytomegalovirus (CMV) retinitis.^147^ ASOs mainly function as expression inhibitors through the RNase H enzyme-mediated mRNA degradation pathway^146^ (Figure 2). Other studies suggested that ASOs might inhibit 5′ end capping and 3′ end polyadenylation once bound with pre-mRNAs, leading to the destabilization of RNAs.^148^ Additionally, it has been reported that ASOs could be designed to bind with the intron-exon boundaries of targeted pre-mRNAs for splicing regulation.^148^^,^^149^

#### siRNA

siRNA refers to a 21- to 23-nt-long double-stranded RNA, usually with two free bases at the 3′ end.^150^ Matured siRNAs formed by cleavage of internalized exogenous long double-stranded RNAs (dsRNAs) or short hairpin RNAs (shRNAs), have been demonstrated to introduce cleavage or degradation on mRNA targets.^151^ Artificially designed siRNAs with perfect base-pair matching can be synthesized and transfected into host cells for gene transcription manipulation. In the cytosol, siRNA duplexes participate in the formation of RNA-induced silencing complex (RISC) with Argonaute 2 protein (Ago2), resulting in separated single strands.^152^ Once the RISC-bound antisense sequences specifically match the target mRNAs, mRNA cleavage is induced by Ago2, followed by RNase-mediated hydrolysis^153^ (Figure 2). Notably, because RISC-bound siRNAs are protected from nuclease degradation, they can render prolonged effects via siRNA recycling and repeated degradation of mRNAs.^154^

#### miRNA mimic or inhibitor

miRNAs were primarily discovered as endogenous ncRNAs involved in RNA-mediated gene silencing in mammalian cells.^155^ RNA polymerase II mediates miRNA transcription in the nucleus by forming primary miRNA (pri-miRNA) transcripts.^156^ These transcripts are cleaved by Drosha and co-factor protein Dgcr8, resulting in precursor miRNAs, namely pre-miRNA.^157^ Once translocated to the cytoplasm and further cleaved by Dicer along with transactivation-responsive RNA-binding protein (Trbp) to form miRNA duplex, one strand of miRNA binds with RISC, leading to translational inhibition or degradation on target mRNA.^158^^,^^159^ Due to their ability to manipulate mRNA abundance, synthetic miRNA mimics or inhibitors have been developed as applicable therapeutic approaches for various diseases. miRNA mimics are synthetic RNA duplexes containing strands identical to those of the corresponding miRNAs, facilitating the restoration or enhancement of miRNA functions.^160^ On the other hand, inhibiting miRNA function can be achieved by using anti-miRNA oligonucleotides (anti-miRs).^161^ Anti-miRs are single-stranded oligonucleotides structurally similar to ASOs, which have been shown to directly bind with the target miRNAs, displaying promising utilizations in miRNA therapeutics.^161^ Currently, phase 2 trials of miRNA mimics for keloid treatment (NCT03601052)^162^ and anti-miRs, known as miravirsen, for hepatitis C virus (HCV) therapy (NCT01200420) have been completed.^163^

#### saRNA

Unlike the gene-silencing oligonucleotides mentioned above, saRNAs are 21-nt double-stranded RNAs that interact with promoters to induce transcriptional activation in an Ago2-dependent manner.^164^^,^^165^ Although the mechanism of saRNAs has not yet been clarified, their therapeutic potential has been investigated.^166^^,^^167^ For instance, hepatocyte nuclear factor 4α (Hnf4α) is a crucial liver-specific transcription factor to mediate hepatocyte differentiation,^168^ liver morphogenesis,^169^ and lipid metabolism.^170^ Liver-specific deletion of *HNF4α* in mice displayed deleterious effects in increasing liver lipid accumulation.^171^ Huang et al. developed saRNA oligo-dendrimers targeting *HNF4A* P1 promoter to enhance *HNF4A* expression. The results showed favorable metabolic profile change with reduced liver TGs and IR improvement in high-fat diet (HFD)-fed rats, indicating that saRNA-mediated *HNF4A* activation may represent a new therapeutic strategy for NAFLD and IR.^172^

### Modifications of synthetic oligonucleotides

To improve specific and effective delivery to target tissues, chemical modifications of synthetic oligonucleotides have been proven as necessary strategies. These modification strategies can be applied to nucleic acid backbone, ribose sugar, and nucleobase singly or in combination to enhance the stability and efficacy of oligonucleotide drugs.^13^ In particular, the modifications on oligonucleotide backbones have involved primarily replacing phosphodiester (PO) linkages with phosphorothioate (PS) linkages. In this process, sulfur atoms are utilized to substitute non-bridging oxygen atoms of the internucleotide phosphate group to increase nuclease resistance.^173^ Balancing the ratio between PO and PS linkages residing in the same oligonucleotide molecule is considered critical to reducing undesired effects such as prolonged retention and compromised target binding.^174^ Notably, Rp and Sp isomers are two configurations for PS linkages. It has been shown that PS linkages with the Sp configuration are more stable than its stereochemical counterparts.^175^ A study team from Wave Life Sciences demonstrated that the DNA region with an (RpSpSp)_3_ core within ASO Gapmer (described below) were more effective than a stereorandom arrangement in leading RNase H1-mediated degradation on target mRNAs.^175^ Moreover, 5′-phosphate terminal modifications were developed to enhance the efficacies of siRNAs, as the phosphorylated 5′ end of the guide strand was found to interact with the middle domain of Ago proteins.^176^ The newly developed 5′-phosphate analogs including 5ʹ-C-methyl, 5ʹ-methylenephosphonate, and 5ʹ-vinylphosphonate are shown to have conformations and steroidal electronic properties similar to those of natural phosphates while displaying resistance against dephosphorylases.^177^^,^^178^ Ribose sugar modifications are commonly designed to substitute the 2ʹ-hydroxyl group on RNA with 2ʹ-*O*-methyl (2ʹ -OMe), 2ʹ-*O*-methoxyethyl (2ʹ-MOE), or 2ʹ-fluoro (2ʹ-F), which have been verified to increase the half-lives of oligonucleotides in plasma and improve their binding affinities^179^^,^^180^^,^^181^^,^^182^ but cannot lead to RNase H activation.^183^^,^^184^ Bridged nucleic acids (BNAs) are featured by a linkage joining the 2ʹ oxygen to 4ʹ carbon between the ribose,^185^ including locked nucleic acid (LNA),^186^ 2ʹ,4ʹ-constrained 2ʹ-*O*-ethyl (constrained ethyl) BNA (cEt),^187^ and 2ʹ-O,4ʹ-C-ethylene-bridged nucleic acid (ENA).^188^ The most commonly used LNA has been found to significantly improve the thermodynamic stability and nucleic acid recognition potential with increased melting temperature.^189^ Further studies have developed alternative chemistries to alter the original DNA or RNA structures, resulting in excellent resistance against various enzymes and unwanted aggregation once linked with charged bioconjugates, such as cationic cell-penetrating peptides (CPPs, described in “other bioconjugations”).^190^^,^^191^ For instance, peptide nucleic acids (PNAs) have aminoethylglycine backbones with acetyl linkers,^192^ while phosphorodiamidate morpholino oligomers (PMOs) have backbones consisting of morpholine rings that bear methylene groups.^193^ In addition, unlocked nucleic acids (UNAs) with unconnected 2ʹ and 3ʹ carbons,^194^ glycol nucleic acids (GNAs) using propylene glycol to alter ribose or deoxyribose,^195^ and tricyclo-DNAs (tcDNAs) with an additional ethylene bridge between the 3ʹ and 5ʹ carbons have also been tested.^196^ Strategies for nucleobase modifications have also been widely investigated. For instance, 2-thiouridine, pseudouridine (Psi), and dihydrouridine have been shown to enhance the thermodynamic stability and gene-silencing efficacy of particular siRNAs/ASOs.^197^

#### ASO

**Figure 3:**
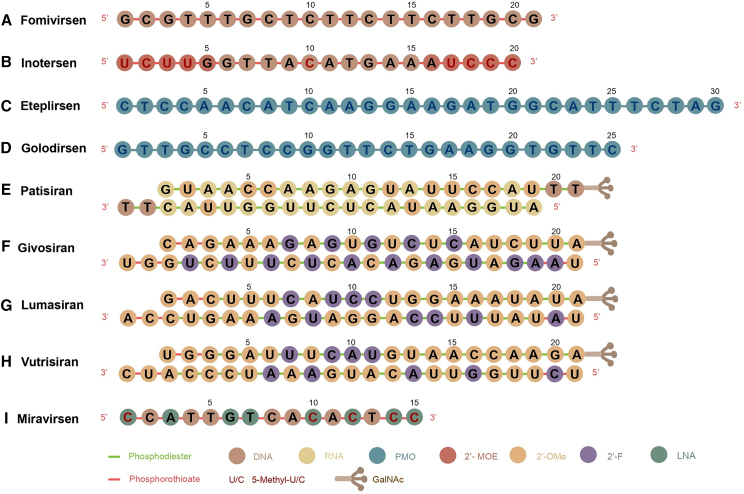
Chemical modifications on major FDA-approved oligonucleotide drugs Chemical modifications of the FDA-approved oligonucleotide drugs: (A) fomivirsen, (B) inotersen, (C) eteplirsen, (D) golodirsen, (E) patisiran, (F) givosiran, (G) lumasiran, (H) vutrisiran, and (I) miravirsen. Circles in different colors refer to different nucleotides and their derivatives, and short lines with different colors refer to phosphodiester or phosphorothioate linkages. PMO, phosphorodiamidate morpholino oligomers; 2ʹ-OMe, 2ʹ-*O*-methyl; 2ʹ-MOE, 2ʹ-*O*-methoxyethyl; 2ʹ-F, 2ʹ-fluoro; GalNAc, *N*-acetylgalactosamine; 5-Methyl-U, 5-methyluridine; 5-Methyl-C, 5-methylcytidine.

The earliest attempts at ASO modification mainly included PS linkage, leading to the advent of FDA-approved fomivirsen^198^ (Figure 3). However, studies have shown that PS may cause compromised interaction between ASO and target mRNA.^199^ As the second generation of ASOs, Gapmer is a short central DNA segment flanked by RNA-based sequences on both sides.^13^ Due to the hybrid structure that is resistant to nuclease and allows modifications on the RNA flanks, Gapmer has been shown to display improved target-binding ability.^200^^,^^201^ Inotersen, utilizing the Gapmer structure to target transthyretin (*TTR*) mRNA for the treatment of hereditary transthyretin (hATTR)-mediated amyloidosis, was successfully developed and approved by the FDA in 2018^202^ (Figure 3). More advanced strategies such as LNA, PNA, and PMO are prevalently adopted in recent ASO design.^203^ Eteplirsen and golodirsen, utilizing PMO technology, were approved by the FDA in 2016 and 2019, respectively, for the treatment of Duchenne muscular dystrophy (DMD)^204^^,^^205^ (Figure 3).

#### siRNA

The aforementioned PS backbone and ribose sugar-modification strategies^180^^,^^181^^,^^182^ have also been widely used in innovating siRNA drugs.^206^ For example, patisiran is a 2ʹ-OMe modified siRNA-based drug approved by the FDA in 2018 to silence mutated *TTR* expression in hATTR liver^207^^,^^208^ (Figure 3). Moreover, combinations of different modifications at specific sites are desirable.^209^^,^^210^^,^^211^ The FDA-approved anti-*ALAS1* RNAi drug givosiran is an example that introduces both 2ʹ-OMe and 2ʹ-F modifications^208^ (Figure 3). Special modification patterns were developed by Alnylam Pharmaceuticals, including standard template chemistry (STC), enhanced stabilization chemistry (ESC), advanced ESC, and ESC-Plus (ESC+). STC pattern was designed as an alternative, with 2ʹ-OMe and 2ʹ-F modifications employed in both siRNA strands except three consecutive 2ʹ-F modifications placed at positions 9, 10, and 11 of the passenger strand and consecutive 2ʹ-OMe modifications placed at positions 11, 12, and 13 of the guide strand.^206^ Additionally, two PS linkages are added at the 3ʹ end of the guide strand. Although the new pattern invested siRNAs with higher stability and affinity, safety remains a significant concern.^212^ To reduce toxicity and further improve stability, fewer 2ʹ-F modifications and four more PS linkages were added to the ESC pattern.^213^ Givosiran, lumasiran, and vutrisiran are successful representatives of the ESC pattern^17^ (Figure 3). Thereafter, Alnylam explored multiple modification design variants by changing the proportion and position of 2ʹ-F and 2ʹ-OMe.^206^ Compared to the former ESC pattern, ESC+ introduced a GNA at position 7 of the guide strand, which was shown to reduce the off-target effects of *N*-acetylgalactosamine (GalNAc) siRNAs.^214^^,^^215^ Now the ESC+ pattern is applied to the development of new drugs, as seen in ALN-HBV02 for treating chronic hepatitis B virus (HBV) infection (NCT03672188) and zilebesiran for treating hypertension (NCT05103332).

#### miRNA mimic or inhibitor

For miRNA-based therapy, modifications including PS, LNA, and 2ʹ-OMe are widely utilized to protect oligonucleotides from RNase-mediated degradation. Because the guide strand in miRNA mimics needs to be recognized by RISC, fewer modifications (such as 2'-F modification) are available, while the passenger strand can be modified (such as 2ʹ-OMe) and linked to bioconjugations (such as cholesterol).^216^ Given that single-stranded anti-miRs are structurally similar to ASOs, most of the chemical modification strategies applied in ASOs could be utilized.^217^^,^^218^^,^^219^^,^^220^^,^^221^^,^^222^^,^^223^ Currently, miravirsen, an anti-miR-122 modified with LNA, was developed by Santaris Pharma for chronic HCV genotype 1 infection treatment^224^ (Figure 3). Studies have shown that by adding LNA modification, anti-miRs significantly antagonized the endogenous miRNAs.^225^^,^^226^ In addition, a variety of different sequences are designed as double-stranded domains or hairpin structures and added on both ends of anti-miR to improve the binding affinity and nuclease stability.^227^ Furthermore, Krützfeldt et al. innovated a special modification combination “antagomir” by using 2′-OMe sugar modification, PS backbone modification, and cholesterol conjugation on the 3′ end.^228^ Thanks to the specific, efficient, and long-lasting gene silencing, antagomir is now widely used in *in vivo* tests.^229^^,^^230^

### Safety issues of synthetic oligonucleotides

The common adverse drug reactions (ADRs) of oligonucleotides reported in various clinical studies include injection-site reactions, headache, fever, and hypersensitivity,^231^ making oligonucleotide-mediated side effects a big concern.

Mechanistically, by base pairing with targeted mRNA sequences, oligonucleotides may cause on-target or off-target toxicities.^232^ On-target toxicities refer to exaggerated intended effect (e.g., too strong silencing of the targeted mRNA) and/or target-gene silencing in unwanted organs.^232^ To avoid such problems, tissue-specific delivery systems are needed, while accurate assessments of tissue-related expression pattern and biological function in disease-relevant cell lines or primary human cells should be conducted in pre-clinical investigations.^233^ On the other hand, off-target toxicities are adverse pharmacological effects caused by undesired silencing on unrelated transcripts.^232^ In terms of this issue, *in silico* screening and *in vitro/in vivo* targeting evaluation are widely used,^234^^,^^235^ while transcriptomics analysis to evaluate hybridization specificity is also suggested.^236^

Other toxicities independent of base pairing can cause inflammation responses, impaired coagulation, and abnormal complement activation, as well as tissue damage in kidney and liver.^232^ For example, most of the earlier generation of siRNA drugs, such as genasense for the treatment of melanoma, were shown to trigger unmethylated cytosine phosphate-guanine (CpG) motif-induced immune stimulation.^237^^,^^238^ ASO-based ISIS2302 targeting intercellular adhesion molecule 1 (*ICAM-1*) was found to inhibit coagulation in cynomolgus monkeys.^239^ To solve these issues, precise determinations of safe concentration and efficiency of oligonucleotides are imperative. Moreover, introducing novel chemical modifications (such as 2ʹ-hydroxyl group substitution and PMO) is currently being tested and applied. Intriguingly, several modification species aiming to increase binding affinity to mRNAs, such as LNAs, may also bring extra risks to off-target toxicities.^240^^,^^241^ Therefore, it is crucial to find the proper kinetics between oligonucleotide drug and its pharmacological target in the particular disease condition.

### Hepatic delivery systems of oligonucleotides

The liver is the largest visceral organ in the body, with a unique circulatory system^142^ where a great number of metabolic targets are susceptible to be regulated by various therapeutic nucleic acids, including oligonucleotides.^15^ To develop effective delivery methods for liver-targeting oligonucleotides in clinical applications, intensive studies have utilized various approaches including chemical modifications, GalNAc conjugates, liposomes, and viral vectors.^15^ To date, the GalNAc-conjugate platform has been proven to be an accessible solution for hepatocyte-targeted oligonucleotides.^242^ Based on the sophisticated chemical modification technologies (STC, ESC, advanced ESC, and ESC+) in combination with GalNAc, Alnylam Pharmaceuticals has innovated a series of FDA-approved RNAi drugs (givosiran, lumasiran, and vutrisiran) and several oligonucleotide candidates currently undergoing clinical trials. On the other hand, lipid nanoparticles (LNPs) could achieve hepatocyte-specific delivery via apolipoprotein E (ApoE)/low-density lipoprotein receptor (LDLR) interaction.^243^^,^^244^ Despite the high transduction efficiency, virus-based delivery approaches are mainly used to demonstrate proof of concept for the therapeutic potential of certain oligonucleotides because of safety concerns.^245^ Collectively, liver-targeting oligonucleotide delivery platforms are becoming more mature and implementable, laying the foundation for the development of oligonucleotide drugs to treat NASH.

#### GalNAc conjugates

The asialoglycoprotein receptor (ASGPR) was discovered as a lectin in rabbits by Gilbert Ashwell and Anatol Morell in 1965.^246^ Galactose was later identified as a terminal sugar residue necessary for ASGPR binding, where the number and arrangement of galactose residues were significantly involved.^247^^,^^248^^,^^249^^,^^250^^,^^251^^,^^252^ By substituting an *N*-acetylamine (AcNH) to the OH group at C-2 position (Figure 2), the galactose derivative GalNAc was shown to be more rapidly endocytosed by hepatocytes at the sinusoidal surface.^253^^,^^254^^,^^255^ It then dissociates from ASGPR upon endosome lumen pH drop, resulting in degradation of GalNAc and membrane recycling of ASGPR.^256^^,^^257^ Rogers and Kornfeld initiated liver-targeted cargo delivery via ASGPR by transferring fetuin glycopeptide-coupled proteins into the rat liver.^258^ Subsequently, researchers sought to deliver different substances into hepatocytes through this pathway, including therapeutic glycolipids,^259^ chemotherapy drugs,^260^ and nucleotides.^261^^,^^262^ Hangeland et al. achieved the successful delivery of an oligodeoxynucleoside methylphosphonate neoglycopeptide conjugate, [YEE (ah-GalNAc) 3]-SMCC-AET-pUmpT7, into human hepatocellular carcinoma cells (HepG2) in 1995.^263^ Since then, the use of GalNAc conjugation to enhance the delivery efficiencies of ASOs and siRNAs has been constantly investigated and optimized.^264^^,^^265^^,^^266^ Prakash et al. developed a triantennary GalNAc-conjugated ASO, improving the potency of hepatocyte-targeted delivery by 10-fold in mice.^267^ Notably, GalNAc conjugated with ASOs or siRNAs now are shedding light on the clinical applications of liver-targeted oligonucleotide drugs. For instance, givosiran was designed to utilize the ESC-GalNAc delivery platform targeting *ALAS1*, a key enzyme gene upregulated in AHP.^17^ Lumasiran and vutrisiran were designed for liver-targeted gene silencing of hydroxyacid oxidase 1 (*HAO1*) in primary hyperoxaluria type 1 (PH1),^268^ and *TTR* in hATTR amyloidosis,^269^ respectively (Figure 3). Meanwhile, Ionis Pharmaceuticals is leading the ongoing ligand-conjugated ASO (LICA) program, which began with the GalNAc conjugation platform developed to achieve liver-targeted inhibition of *TTR* mRNA and apolipoprotein C3 (*APOC3*) mRNA.^144^^,^^270^^,^^271^

#### Other bioconjugations

In addition to GalNAc, other bioconjugations, including lipids, peptides, aptamers, and antibodies, have also been tested. Cholesterol and its derivatives, linked with the 3′ ends of passenger stands, are considered some of the most attractive lipid conjugates. Cholesterol-conjugated siRNAs have been shown to exhibit stronger binding to lipoproteins to enhance cellular transportation and uptake.^272^ Moreover, long-chain FAs and α-tocopherol are used to enhance siRNA delivery efficiencies to the liver.^272^^,^^273^ Peptide conjugates, such as CPPs, which are short cationic and/or amphipathic peptides typically equipped with fewer than 30 amino acids, have demonstrated the ability to cargo different molecules and traverse biological membranes via peptide-mediated uptake mechanisms.^274^ Therefore, CPPs are usually introduced to enhance the bioavailability and the target tissue uptake of oligonucleotides.^275^ Aptamers and antibodies are potentially optimal conjugates for delivering oligonucleotides into other cells and tissues due to their specific interactions with non-hepatocyte surface receptors.^276^^,^^277^

#### Lipid nanoparticles

LNPs, utilizing physiologically relevant lipids as nanocarriers, are considered low in toxicity and biocompatible.^278^ It has been demonstrated that LNPs can be internalized via the endocytosis process followed by endosomal escape to facilitate the release of oligonucleotides in the cytosol.^279^ LNPs typically consist of four lipid components: distearoylphosphatidylcholine (DSPC), cholesterol, ionizable cationic lipid, and polyethylene glycol (PEG)-lipid. DSPC and cholesterol are related to LNP structure formation.^280^ Ionizable cationic lipids are used to improve membrane fusion efficiencies and avoid immune responses via low surface charge at physiological pH,^281^ while PEG-lipids are added to control particle size and prevent aggregation.^282^^,^^283^ LNP-encapsulated siRNA cargoes have been shown to accumulate in hepatocytes, KCs, and sinusoids, while the strongest gene-silencing effect is typically achieved in hepatocytes.^284^ LNPs can be further modified to enhance binding specificities toward hepatocytes^244^ and HSCs^285^ by conjugating with GalNAc and vitamin A, respectively.

Intensive studies have shown the therapeutic potential of LNP-encapsulated oligonucleotides delivered to the liver for treating various diseases.^282^ For instance, the aforementioned patisiran is an approved LNP-RNAi drug^207^ that utilizes the ionizable cationic lipid dilinoleylmethyl-4-dimethylaminobutyrate (DLin-MC3-DMA) and results in more than two orders of silencing effect compared to the original 1,2-dilinoleyloxy-*N*,*N*-dimethyl-3-aminopropane (DLinDMA).^286^ The PEG-lipid in this system is the shorter dimyristyl (C14) chain, which has been shown to mitigate the negative impacts of PEG shielding on siRNA silencing *in vivo*.^287^ Moreover, clinical trials are under way for the LNP-encapsulated siRNA ARB-001467 for treating HBV infection (NCT02631096) and BMS-986263 for treating liver fibrosis (NCT03420768).^288^ Notably, LNP-encapsulated siRNAs targeting high-mobility group box 1 (*HMGB1*)^289^ and methylation-controlled J protein (*MCJ*)^290^ have been tested in pre-clinical NASH models, respectively. In addition, LNPs have demonstrated the ability to deliver miRNA into the liver. For instance, an miR-30a-5p mimic was encapsulated into lipid-protamine-hyaluronic acid (LPH) nanoparticle modified with HSC-targeting aminoethyl anisamide (AEAA) to treat liver fibrosis in mice.^291^

#### Viral vectors as proof-of-concept research approaches

Since 1990, when retrovirus was first applied for clinical gene therapy of adenosine deaminase (ADA)-deficient severe combined immunodeficiency (ADA-SCID),^292^ viral vectors for the delivery of nucleotide agents have rapidly developed. Lentiviruses (LVs), adenoviruses (AdVs), and adeno-associated viruses (AAVs) are three major types of viral vehicles currently used.^293^ Due to relatively lower relevance to human diseases, compromised immunogenicity, and cytotoxicity, AAVs are nowadays considered safer viral vectors for *in vivo* expression of oligonucleotide molecules.^294^ In addition, tissue tropism varies greatly in different AAV serotypes,^295^ among which AAV8 has been shown to be a reliable vector to transduce for hepatocytes.^296^ Therefore, despite the controversies on AAVs as a suitable system for NASH therapy, this delivery platform has been intensively utilized in therapeutic target discovery. By introducing shRNA or pri-miRNA expressing cassettes that are driven by hepatocyte-specific promoters into viral vectors, AAVs can be used as a potent liver-targeted delivery approach for mRNA-modulating regions (e.g., siRNAs, miRNAs, and anti-miRNAs). For example, AAV-anti-miR-20b was shown to slow NAFLD progression by upregulating FAO and attenuating IR.^297^ AAV6-mediated *in vivo* expression of the shRNA against pyruvate kinase L/R (*PKLR*) was reported to lower L-type pyruvate kinase expression in the liver of mice fed a high-fat and sucrose (HF/HS) diet, leading to alleviated IR and reduced liver steatosis.^298^ AAV8 harboring shRNA against *SMS1* (sphingomyelin synthase 1) was administered in mice fed a high-fat/cholesterol diet (HFHCD), resulting in lowered expression of pro-inflammatory factors and collagen type III α1.^299^

## Current status of research in oligonucleotide drug developments for NASH

**Figure 4:**
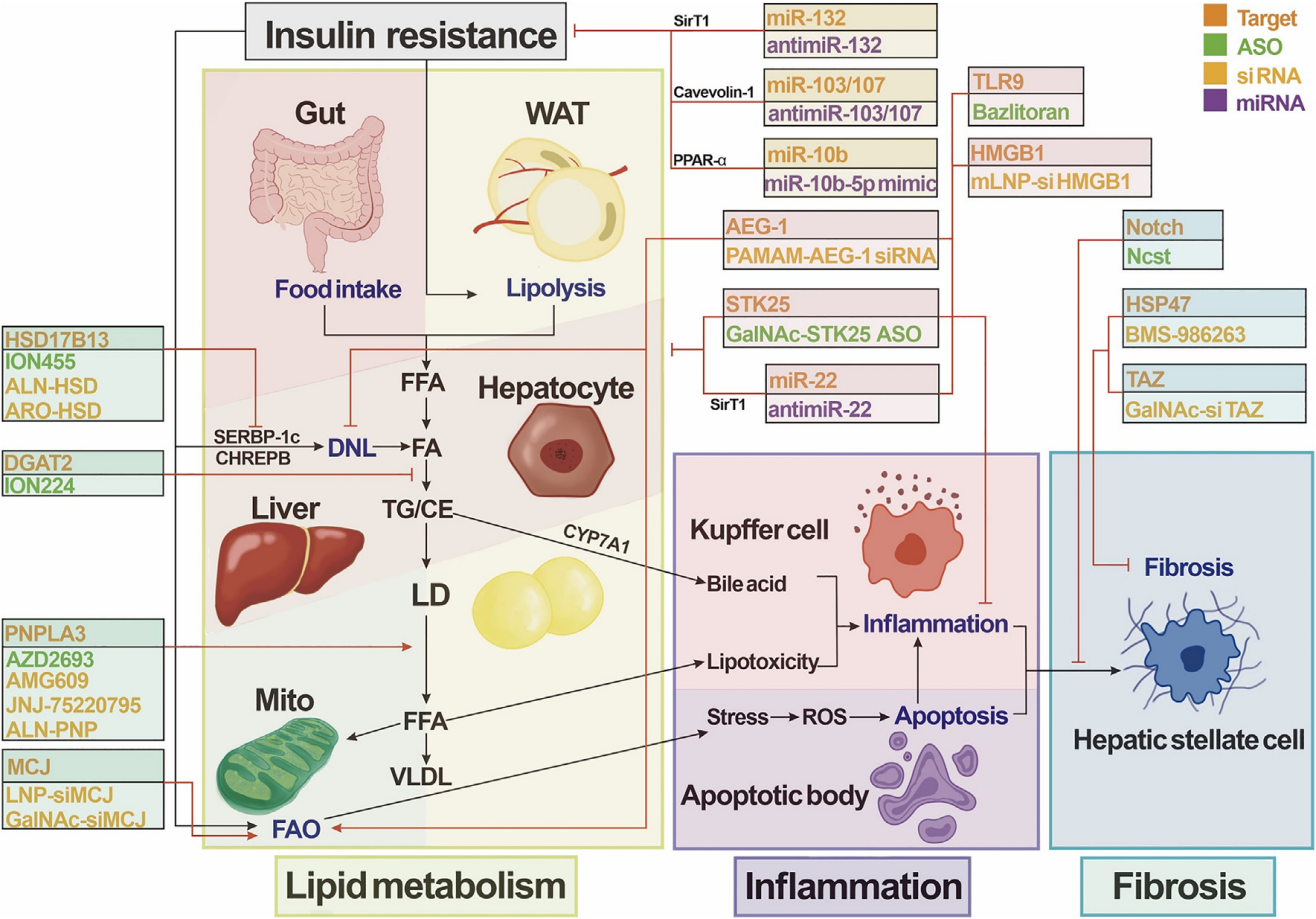
Oligonucleotide drugs for NASH Oligonucleotide drugs for nonalcoholic steatohepatitis (NASH) are now in development, including the projects now closed. Drugs are categorized according to their targets in the NASH pathogenesis. TLR9, Toll-like receptor 9; HSP47, 47-kDa heat-shock protein; STK25, serine/threonine protein kinase 25; HMGB1, high-mobility group box 1; TAZ, transcriptional co-activator with PDZ-binding motif; HSD17B13, 17β-hydroxysteroid dehydrogenase 13; DGAT2, diacylglycerol acyltransferase 2; PNPLA3, patatin-like phospholipase domain-containing 3; MCJ, methylation-controlled J protein; SirT1, silent information regulator 1; RTK, receptor tyrosine kinase; FAS, fatty acid synthase; AEG-1, astrocyte elevated gene 1; SREBP-1c, sterol-regulatory element binding protein 1c; CHREBP, carbohydrate response element binding protein; CYP7A1, cholesterol 7α-hydroxylase; PPAR, peroxisomal proliferator-activated receptor; Ihh, Indian hedgehog; WAT, white adipose tissue; FFA, free fatty acid; DNL, *de novo* lipogenesis; TG, triglyceride; CE, cholesteryl ester; LD, lipid droplet; VLDL, very-low-density lipoproteins; FAO, fatty acid oxidation; mito, mitochondria; ROS, reactive oxygen species; HSC, hepatic stellate cell.

To date, various chemical compounds or small peptides have been developed to modulate a large number of potential therapeutic targets for NASH. Most of these target proteins mainly serve as enzymes or ligands/receptors, leaving insufficient pharmacological approaches applicable for other “less druggable” targets. Alternatively, emerging oligonucleotides are expected to modulate these targets through transcriptional regulation, offering new hopes for NASH treatments (Figure 4). In this context, we have summarized oligonucleotide therapeutics in NASH clinical trials and major pre-clinical studies (Tables 2 and 3).

**Table 2:** Oligonucleotide therapeutics for NASH in clinical trials

Name (company)	Targeted gene	Targeting agent	Disease	Latest status	ClinicalTrials.gov identifier	Reference
AZD2693 (Ionis Pharmaceuticals)	*PNPLA3*	ASO/ASO-GalNAc conjugate	NASH	phase 2	NCT04483947	Ionis Pharmaceuticals^306^
ION224 (Ionis Pharmaceuticals)	*DGAT2*	ASO/ASO-GalNAc conjugate	NASH	phase 2	NCT04932512	Ionis Pharmaceuticals^311^
ALN-HSD (Alnylam Pharmaceuticals)	*HSD17B13*	siRNA/(ESC+)-GalNAc conjugate	NASH	phase 2	NCT05519475	Regeneron Pharmaceuticals^352^
ARO-HSD (Arrowhead Pharmaceuticals)	*HSD17B13*	siRNA/siRNA-GalNAc conjugate	NASH	phase 1	NCT04202354	Mak et al.^353^
ION455 (Ionis Pharmaceuticals)	*HSD17B13*	ASO/ASO-GalNAc conjugate	NASH	phase 1	NCT05143905NCT05560607	Ionis Pharmaceuticals^354^
BMS-986263 (Bristol Myers Squibb)	*HSP47*	retinoid-conjugated LNP containing siRNA	NASH	phase 2	NCT04267393	Lawitz et al.^288^
AMG 609 (Amgen)	*PNPLA3*	siRNA-GalNAc conjugate	NAFLD	phase 1	NCT04857606	N/A
JNJ-75220795 (Arrowhead Pharmaceuticals)	*PNPLA3*	TRIM platform^419^	fatty liver disease	phase 1	NCT04844450NCT05039710	Arrowhead Pharmaceuticals^363^
ALN-PNP (Alnylam Pharmaceuticals)	*PNPLA3*	siRNA (in ESC+/GalNAc platform)	NASH	phase 1	NCT05648214	N/A
AZD4076 (Regulus Therapeutics)	*miR-103/107*	GalNAc-conjugated anti-miRNA	NAFLD/T2D	phase 1/2a	NCT02826525	Regulus Therapeutics^401^
			NASH	phase 1	NCT02612662	

*PNPLA3*, patatin-like phospholipase domain-containing 3; *DGAT2*, diacylglycerol acyltransferase 2; *HSD17B13*, 17β-hydroxysteroid dehydrogenase 13; *HSP47*, 47-kDa heat-shock protein.

**Table 3:** Oligonucleotide therapeutics in pre-clinical studies

Name (company)	Targeted gene	Targeting agent	Targeting cell	Disease	Animal model	Reference
GalNAc-Stk25ASO (Sprint Bioscience AB)	*STK25*	ASO/ASO-GalNAc conjugate	hepatocyte	NASH/T2D	murine	Cansby et al.^316^
*MST3*-targeting ASO	*MST3*	ASO	hepatocyte	NAFLD	murine	Caputo et al.^318^
GalNAc-ASO-*ADGRF1*	*ADGRF1*	ASO-GalNAc conjugate	hepatocyte	NAFLD	murine	Wu et al.^320^
GalNAc-ASO-*PCSK7*	*PCSK7*	ASO-GalNAc conjugate	hepatocyte	NAFLD	murine	Sachan et al.^323^
AVO101 (Avogadro Pharmaceuticals)	*TLR9*	ASO	N/A	NASH	primate	Shepard et al.^329^
NCST	*nicastrin*	2′-O-MOE modified ASO	hepatocyte	NASH	murine	Zhu et al.^332^
LNP-siMCJ/GalNAc-siMCJ	*MCJ*	LNP/siRNA-GalNAc conjugate	hepatocyte	NASH	murine	Barbier-Torres et al.^290^
PAMAM-AEG-1si	*AEG-1*	nanoplexes conjugating PAMAM-PEG-Gal	hepatocyte	NASH	murine	Srivastava et al.^370^
GalNAc-siTAZ	*TAZ*	GalNAc-siRNA	hepatocyte	NASH	murine	Wang et al.^375^
mLNP-siHMGB1	*HMGB1*	mannose-modified siRNA loaded LNP	Kupffer cell	NASH	murine	Zhou et al.^289^
OLX702A (OliX Pharmaceuticals)	N/A	asiRNA-GalNAc conjugate	N/A	NASH	primate	OliX Pharmaceuticals^385^
anti-miR-132 (Regulus Therapeutics)	miR-132	2′-F and 2′-O-Me modified anti-miRNA	hepatocyte	NASH	murine	Papazyan et al.^394^
RES-010 (Resalis Therapeutics)	miR-22	LNA modified anti-miRNA	hepatocyte	NASH/NAFLD	murine	Thibonnier et al.^406^
anti-miR-33	miR-33	amido-bridged nucleic acids (AmNAs)^420^	hepatocyte	NASH	murine	Miyagawa et al.^413^
MiR-10b-5p mimic (RosVivo Therapeutics)	miR-10b-5p	miRNA mimic	N/A	NAFLD/T2D/obesity/GI	N/A	RosVivo Therapeutics^418^

*STK25*, serine/threonine protein kinase 25; *MST3*, mammalian sterile 20-like 3; *ADGRF1*, adhesion G-protein-coupled receptor F1; *PCSK7*, proprotein convertase subtilisin/kexin type 7; *TLR9*, Toll-like receptor 9; *MCJ*, methylation-controlled J protein; *AEG-1*, astrocyte elevated gene 1; *TAZ*, transcriptional co-activator with PDZ-binding motif; *HMGB1*, high-mobility group box 1.

### ASO

#### Patatin-like phospholipase domain-containing protein 3

Patatin-like phospholipase domain-containing 3 (*PNPLA3*) encodes Pnpla3 protein with TG hydrolase activity in hepatocytes.^300^ Amino acid substitution from isoleucine (I) to methionine (M) at position 148 (I148M) has been reported to have a robust association with various liver metabolic diseases including steatosis and fibrosis/cirrhosis,^300^ probably due to reduced enzymatic activity.^301^ Further studies have shown that ubiquitylation and proteasome-mediated Pnpla3 degradation were impaired by the I148M substitution, leading to the accumulation of mutated Pnpla3 in LDs and enhancing steatosis.^302^^,^^303^ Moreover, the overexpression of Pnpla3 I148M in an NAFLD mouse model upregulated the transcription of several marker genes involved in UPR and induced the accumulation of oxidized glutathione, suggesting its association with ER and oxidative stress.^304^ In a pre-clinical study, S-cEt-modified 16-mer ASOs were screened for optimal targeting on the mouse *PNPLA3* gene.^305^ The resultant ASO was further modified by 5′ end conjugation with triantennary GalNAc.^305^ The potency of anti-PNPLA3 ASO-GalNAc in improving NAFLD conditions caused by mutated PNPLA3, including liver fibrosis, was proven.^305^ Furthermore, ASO/ASO-GalNAc conjugate AZD2693(ION839) was innovated by Ionis Pharmaceuticals and AstraZeneca to inhibit *PNPLA3* expression.^306^ A phase 2 study of AZD2693 with NASH patients carrying Pnpla3 I148M has been launched (NCT05809934).

#### Diacylglycerol acyltransferase 2

Diacylglycerol acyltransferase 2 (Dgat2) catalyzes TG synthesis from diacylglycerol and fatty acyl CoA as substrates.^64^ *DGAT2-*knockout mice were found dead soon after birth due to lipopenic phenotypes, such as dysregulated energy metabolism and impaired skin barrier function.^307^ Given that TG accumulation is considered one of the key steps in NAFLD pathogenesis,^27^ ASO-mediated *DGAT2* silencing was developed. Results showed that ASOs targeting *DGAT2* significantly reduced hepatic lipid storage in rats, accompanied by lowered expressions of lipogenic genes (*SREBP1c*, *ACC1*, *SCD1*, and *mtGPAT*) and elevated expressions of oxidative/thermogenic genes (*CPT1* and *UCP2*).^308^ A parallel study showed administrations of ASOs targeting *DGAT2* in HFD-fed mice and ob/ob mice both efficiently reduced liver Dgat2, resulting in lowered intrahepatic TG level and attenuated hyperlipidemia, as well as reduction of hepatic steatosis.^309^ However, further studies using the NASH mouse model induced by a diet deficient in methionine and choline (MCD) showed that ASO-mediated *DGAT2* silencing aggravated hepatic inflammation and fibrosis via elevated FFA-associated oxidative stress,^310^ indicating critical roles of non-TG lipid products initiating hepatotoxicity in NASH progression. ION224 is a *DGAT2*-targeting ASO innovated by Ionis Pharmaceuticals.^311^ The phase 2 clinical trial of ION224 (NCT04932512) was completed with positive results showing improvement in NAS score without worsening fibrosis in NASH patients.^312^

#### Other targets in lipid metabolism

LD-associated protein serine/threonine protein kinase 25 (Stk25) has been demonstrated to play an inhibitory role in regulating lipid oxidation and insulin sensitivity.^313^ Biopsy data showed a positive correlation between Stk25 abundance and fat content in human livers.^314^ In addition, *STK25* transgenic mice displayed a dramatic increase in liver lipid deposition, hepatic IR, and steatohepatitis.^314^ Consistently, repressed NASH symptoms including liver steatosis and oxidative damage were found in *STK25*-knockout mice.^315^ Cansby et al. designed a triantennary GalNAc-conjugated ASO for hepatocyte-targeted *STK25* silencing (GalNAc-*STK25* ASO), which displayed alleviated NASH symptoms in mice under chronic exposure of dietary lipids without obvious systemic toxicity or local tolerability concerns.^316^ Currently, Sprint Bioscience and Gothenburg University are conducting GalNAc-*STK25* ASO for NASH and T2D treatment in humans.

Mammalian sterile 20-like 3 (Mst3, also known as Stk24) is another LD-associated protein closely related to Stk25.^317^ Chemical modified ASOs targeting *MST3* have also shown the capacity to ameliorate diet-induced NAFLD, including the reduced oxidative stress and ER stress biomarkers (4-hydroxynonenal, 8-oxoguanine, KDEL, and CHOP) in mouse livers.^318^

Adhesion G-protein-coupled receptor F1 (Adgrf1) belongs to the G-protein-coupled receptor (GPCR) family.^319^ Recent studies found that Adgrf1 acted as an upstream regulator of Scd1.^320^ Moreover, two GalNAc-conjugated ASO-*ADGRF1*s that bind to different regions of *ADGRF1* mRNA have been found to improve glucose homeostasis, alleviating lipid abundance and liver damage in HFD-fed *ADGRF1*-overexpressed mice.^320^

Proprotein convertase subtilisin/kexin type 7 (*PCSK7*) encodes Pcsk7 as a transmembrane protease,^321^ whose single-nucleotide variation (rs236918) is linked with dyslipidemia and liver damage in NAFLD patients.^322^ The recent studies led by Sachan et al. have shown that GalNAc-ASO selected to target *PCSK7* mRNA had the ability to accelerate the recovery of high-fat/fructose/cholesterol (HFFC) diet-induced mice exhibiting hepatic steatosis.^323^

#### Toll-like receptor 9

By serving as pattern-recognition receptors (PRRs), Toll-like receptors (TLRs) were demonstrated to recognize unwanted or mislocated DNA fragments, such as unmethylated CpG-DNA motifs from bacteria or virus genome, to initiate tissue inflammation.^324^ Meanwhile, a substantial amount of mitochondrial DNA (mtDNA) was also found in the plasma of NASH patients as well as HFD-fed mice,^325^ where mtDNA was shown to be released into extracellular milieu from injured hepatocytes.^326^ In line with these findings, the mRNA level of *TLR9* (a member of the TLR family) was reported to increase in the livers of NASH patients and atherogenic diet-fed mouse models.^327^ Moreover, pro-inflammatory cytokines in the liver were demonstrated to be mediated by activated Tlr9 along within NASH progression, where Tlr9 antagonist IRS954 could block this process.^325^ *TLR9* knockout led to less liver steatosis, fibrosis, and IR in mice fed with choline-deficient/amino acid-defined (CDAA) diet, probably due to suppressed interleukin-1β and nuclear factor κB (NF-κB) signaling.^328^ All of the above data strongly suggested critical roles of Tlr9 in NASH progression. AVO101, a phase 2-ready *TLR9* ASO, was developed by Shepard et al. to display elevated adiponectin, lowered weight, and reduced NASH symptoms in a primate obesity model.^329^

#### Notch signaling pathway

The Notch signaling pathway is a conserved cellular process well known to be involved in organ formation and morphogenesis.^330^ Under physiological conditions, the Notch pathway was found to be required for bile duct development in nonparenchymal cells but inactive in hepatocytes.^331^ Interestingly, positive correlations between Notch activity in hepatocytes and NASH progression were observed in patients and diet-induced mouse NASH models.^332^ In addition, forced Notch activation was shown to promote secretion of the fibrogenic factor osteopontin (Opn), leading to the activation of HSC-mediated fibrosis.^332^ γ-Secretase is an enzyme catalyzing Notch intramembrane proteolysis to facilitate downstream reactions.^333^ Therefore, various approaches to inhibit γ-secretase have been considered to treat NASH. Given that the commonly used γ-secretase inhibitor (GSI) was found to cause goblet cell metaplasia,^334^ a liver-selective ASO to target *NCST* (the gene encoding one of the γ-secretase complex subunits for ligand-dependent Notch activation) was developed.^332^ Results showed suppressed HSC activation and collagen deposition along with lowered body weight and adiposity in mice.^332^ Moreover, the absence of intestinal toxicity during *NCST* ASO administration indicated its safety via specific targeting of the inappropriately activated Notch signaling in hepatocytes.^332^

#### Long non-coding RNAs

The essential roles of ncRNAs in NAFLD/NASH pathogenesis has been elucidated in recent studies.^335^ Long-ncRNAs (lncRNAs) are large ncRNA transcripts (longer than 200 nt), which are involved in post-transcriptional regulation by directly interacting with proteins or sponging miRNAs (protecting target mRNAs from miRNA binding and degradation).^336^^,^^337^

As a multi-functional lncRNA, nuclear paraspeckle assembly transcript 1 (NEAT1) has been demonstrated as a therapeutic target in several disease conditions. For instance, ASO-based NEAT1 silencing has been utilized in preventing post-stroke LD agglomeration.^338^ Since the level of this lncRNA was found to upregulate in NAFLD and liver fibrosis patients,^339^^,^^340^ silencing NEAT1 by shRNA or siRNA was shown to suppress liver fibrosis and inflammation probably through disrupting the binding with miR-122 and miR-506.^340^^,^^341^ Other studies reported reduced lipid accumulation by shRNA-mediated NEAT1 silencing by derepressing miR-146a-5p and miR-212-5p.^339^^,^^342^ These results indicated that NEAT1 is a promising lncRNA target for ASO-based NAFLD treatment through multi-target regulation.

Given that NASH is highly related to metabolic disorders including IR, diabetes, and diabetic complications, ASO-mediated treatments targeting metabolism-regulatory lncRNAs are thought to confer potential benefits to treat this syndrome.^343^ AstraZeneca developed a Glp-1-conjugated ASO targeting lncRNA metastasis-associated lung adenocarcinoma transcript 1 (MALAT1), to achieve pancreatic β cell-specific oligonucleotide uptake for treating diabetes,^344^^,^^345^^,^^346^ whose application could be transferred to improve dysregulated liver metabolism in NASH.

### siRNA

#### 17β-Hydroxysteroid dehydrogenase 13

Like *PNPLA3* and *STK25*, 17β-hydroxysteroid dehydrogenase 13 (*HSD17B13*) also encodes an LD-associated protein mainly expressed in hepatocytes.^347^ Both the protein and mRNA levels of this gene were observed to be upregulated in human NAFLD liver samples.^348^^,^^349^ Additionally, individuals carrying *HSD17B13* loss-of-function variant (rs72613567: T/A) were found to have reduced risks of NASH and cirrhosis.^350^^,^^351^ Furthermore, AdV-mediated overexpression of human *HSD17B13* led to a fatty liver phenotype in mice,^348^ highlighting its role in promoting NAFLD/NASH pathogenesis. ALN-HSD, a GalNAc-conjugated siRNA, was developed by ESC+ GalNAc-conjugate technology to silence *HSD17B13* expression.^352^ A phase 2 clinical study of subcutaneously administered ALN-HSD for NASH therapy (NCT05519475) is currently led by Alnylam Pharmaceuticals. Meanwhile, ARO-HSD (GSK4532990) siRNA developed by Arrowhead Pharmaceuticals has completed the phase 1 clinical trial (NCT04202354), showing good tolerance with lowered hepatic *HSD17B13* expression as well as decreased serum ALT level in NASH patients.^353^ In addition, Ionis Pharmaceuticals and AstraZeneca developed ION455 (AZD7503) based on LICA ASO targeting *HSD17B13* and is currently launching phase 1 studies (NCT05143905 and NCT05560607).^354^

#### 47-kDa heat-shock protein

The 47-kDa heat-shock protein (*HSP47*) encodes an ER-resident chaperone, which binds to and stabilizes collagens/procollagens via Gly-Xaa-Arg repeats on triple-helical procollagen.^355^^,^^356^ Abnormalities in Hsp47 function have been thought to be associated with tissue fibrosis, such as CCl_4_-induced liver fibrosis and bleomycin-induced pulmonary fibrosis.^357^^,^^358^ Sato et al. showed successful HSC-specific delivery of siRNA targeting rat *HSP47* homolog through vitamin A-coupled liposomes, which alleviated liver fibrosis and resolved collagen deposition in multiple liver disease models induced by dimethylnitrosamine (DMN), CCl_4_, and bile duct ligation, respectively.^285^ Encouraged by these results, *HSP47* siRNA encapsulated in HSC-targeting vitamin A-coupled liposomes were tested in other organs, displaying dampened tissue fibrosis in pancreas, lung, lacrimal glands, and skin.^359^^,^^360^^,^^361^^,^^362^ BMS-986263 (ND-L02-s0201), a retinoid-conjugated LNP encapsulating HSP47 siRNA, has been used to target HSC-mediated liver fibrosis (NCT02227459) and myofibroblast-mediated idiopathic pulmonary fibrosis (IPF) (NCT03538301). It has been shown that fibrosis scores (METAVIR and Ishak) were significantly downregulated in HCV-infected patients with advanced liver fibrosis (NCT03420768).^288^ As of the latest update, a phase 2 clinical trial evaluating the safety and effectiveness of BMS-986263 in NASH patients with compensated cirrhosis is under way (NCT04267393).

#### PNPLA3

As mentioned earlier, the *PNPLA3* I148M variant has been demonstrated as one of the key factors causing hepatocyte lipid accumulation.^303^ AMG 609 is essentially an siRNA that selectively targets the mutated allele. A phase 1 clinical trial evaluating the safety, tolerance, and liver fat changes upon subcutaneously administered AMG 609 has been launched (NCT04857606). Meanwhile, other siRNA drug candidates, JNJ-75220795 (ARO-PNPLA3)^363^ and ALN-PNP, designed for reducing *PNPLA3* expression, are also undergoing phase 1 trials for NASH treatment (NCT04844450, NCT05039710, and NCT05648214).

#### Methylation-controlled J protein

MCJ (also called DnaJC1), located in the mitochondrial inner membrane, was identified as a co-chaperone to inhibit the functions of electron transfer chain (ETC) complex I.^364^ As the ETC serves as the outlet for products of FA β-oxidation, an excessive amount of MCJ may contribute to NAFLD development via abnormally increased FA accumulation.^52^ In fact increased MCJ expression has been reported in NAFLD patients, while reduction of liver steatosis and fibrosis were observed in MCJ-deficient mouse NASH models.^290^ In addition, loss of MCJ was shown to increase FA consumption by promoting biogenesis of respiratory supercomplexes,^364^^,^^365^ leaving electron leakage unchanged.^364^^,^^365^^,^^366^^,^^367^ Since the increase in ROS production from hyperactivated ETC normally impairs mitochondria and aggravates the tissue damage, it is believed that reduction of *MCJ* expression might be a feasible strategy to prevent NAFLD progression.^368^ LNP-siRNA targeting *MCJ* (LNP-si*MCJ*) was shown to result in reduced lipid accumulation, fibrosis, and hepatocyte damage in several NASH models mimicking different disease conditions.^290^ GalNAc-siRNA targeting *MCJ* (GalNAc-si*MCJ*) was also tested to achieve comparable therapeutic effects.^290^

#### Astrocyte elevated gene 1

Previous studies have shown the stimulatory roles of astrocyte elevated gene 1 (*AEG-1*) in the NF-κB pathway to induce inflammation in hepatocytes and macrophages.^369^ Srivastava et al. showed that Aeg-1 protein levels were significantly overexpressed in biopsy samples from NASH patients.^370^ In addition, spontaneous NASH-related pathological changes were observed in transgenic mice with hepatocyte-specific overexpression of *AEG-1*, whereas hepatocyte-specific *AEG-1* knockout was shown to protect mice from HFD-induced NASH.^370^ The versatile functions of Aeg-1 in promoting NASH may be attributed to enhanced DNL and inflammation as well as downregulated FAO in the liver.^370^ Previously validated liver-targeted nanoplexes composing of poly-amidoamine (PAMAM) dendrimers, PEG, and lactobionic acid (PAMAM-PEG-Gal)^371^ were applied to encapsulate and deliver siRNAs that specifically silence *AEG-1* (PAMAM-AEG-1si) in the HFD-induced mouse model, resulting in a significant alleviation of liver damage and downregulated serum AST/ALT, liver weight, and TG/cholesterol levels.^370^

#### Transcriptional co-activator with PDZ-binding motif

Transcriptional co-activator with a PDZ-binding motif (*TAZ*), encoding a transcriptional co-activator sharing homology with Yes-associated protein (Yap), was found to bind to the PPXY motif through its WW domain.^372^ TAZ is considered to be related to mesenchymal differentiation and development of multiple organs.^373^ Wang et al. observed elevated TAZ in the livers from NASH patients and MCD-induced murine models.^374^ In addition, AAV8-mediated liver-specific *TAZ* silencing was shown to reduce hepatic inflammation, hepatocyte death, and fibrosis in a NASH mouse model through repression of Indian hedgehog (Ihh)-mediated fibrogenic gene activation in HSCs.^374^ In the follow-up study, the same research group utilized *TAZ* siRNA conjugated with GalNAc (GalNAc-si*TAZ*) for therapeutic study. The results showed that GalNAc-si*TAZ* was able to prevent or even reverse NASH progression.^375^

#### High-mobility group box 1

Hmgb1 is known as a damage-associated molecular pattern (DAMP) released from nucleus in fat-laden hepatocytes and activated KCs to initiate the activation of the liver pro-inflammatory response as well as fibrosis.^376^^,^^377^^,^^378^ Plasma Hmgb1 level was found to be elevated in a diet-induced NASH mouse model^379^ and positively correlated with the severity of liver fibrosis in NASH patients.^380^ Salvianolic acid B (SalB), a compound that inhibits Hmgb1 nuclear translocation and release, was demonstrated to protect against NAFLD in rats.^381^ Zhou et al. developed a stable mannose-modified LNP delivery system carrying *HMGB1*-siRNA (mLNP-si*HMGB1*) to achieve specific *HMGB1* silencing in KCs via mannose receptors on their surfaces.^289^^,^^382^ The results showed that mLNP-si*HMGB1* reduced Hmgb1 protein in the liver, shifted KCs to M2 phenotype, attenuated fibrosis, and restored liver function in the NASH mouse model.^289^

#### OLX702A (by OliX Pharmaceuticals)

OLX702A is a GalNAc-conjugated asymmetric siRNA (asiRNA) drug with fewer off-target and side effects than other siRNAs.^383^ It was innovated by OliX Pharmaceuticals to target NASH-related genes found in a human NASH genome-wide association study (GWAS).^384^ The administration of OLX702A was shown to significantly reduce liver fat content in a non-human primate NASH model.^385^

#### Long non-coding RNAs

lncRNA HULC (highly upregulated in liver cancer) expression was found to be upregulated in HFD-induced rat models.^386^ Shen et al. demonstrated that siRNA plasmid targeting HULC *in vivo* significantly reduced lipid deposition, fibrosis, and hepatocyte apoptosis in the NAFLD rat model through the p38 mitogen-activated protein kinase (MAPK) and c-Jun N-terminal kinase (JNK) pathways.^386^ Meanwhile, siRNAs targeting other metabolism-related lncRNAs can be potentially utilized in NASH treatments, such as the siRNA designed for silencing lncRNA NONRATT021972, which was shown to alleviate diabetic neuropathy in T2D rat models.^387^^,^^388^^,^^389^

### miRNA mimic or inhibitor

#### MicroRNA-132

miR-132 levels were found to be significantly increased in NAFLD patients and murine NASH models, while transgenic mice with overexpressed miR-132 exhibited liver steatosis and hyperlipidemia.^390^ miR-132 was first demonstrated to inhibit Sirt1 expression through directly binding to *SIRT1* 3ʹ-untranslated region (UTR) in adipocytes.^391^ Sirt1 has been reported to regulate various transcription factors involved in inflammation, lipid metabolism, and insulin secretion (e.g., p53, NF-κB, PPAR-α, PPAR-γ, PPAR-γ co-activator 1α [Pgc-1α], and liver X receptor [Lxr]) via its deacetylase activity in specific sites.^392^^,^^393^ Therefore, anti-miR-132-mediated *SIRT1* derepression could be applied as a potential approach in intervening metabolism-related diseases including NAFLD. Studies have shown that diet-induced obese (DIO) mice treated with anti-miR-132 displayed resolved liver steatosis as well as reduced liver FFA and serum LDL/VLDL.^390^ The above data collectively suggest that the downregulation of miR-132 has the potential to impede NASH progression. Regulus Therapeutics tested oligonucleotide-based miR-132 antagonists in DIO, choline-deficient high-fat diet (CDHFD), and amylin liver NASH (AMLN) models, showing promising efficacies in treatments.^394^

#### MicroRNA-103/107

Differing by only one nucleotide,^395^ miR-103 and miR-107 paralogs exist within the intron region of *PANK*, which encodes the pantothenate kinase (Pank).^396^ They were shown to be co-transcribed with this gene to regulate several target mRNAs involved in lipid and pyruvate metabolic pathways.^396^ Studies have shown that the levels of these two miRNAs were significantly upregulated in the livers of obese mice with steatosis,^397^ which induce impaired glucose homeostasis and insulin sensitivity by inhibiting the expression of cavevolin-1,^398^ a factor known to enhance insulin receptor signaling.^399^^,^^400^ Therefore, anti-miR-103/107 could function as an insulin sensitizer. A study conducted by Regulus Therapeutics showed that the administration of anti-miR-103/107 reduced TG level and liver steatosis in mice.^401^ Clinical trials of RG-125 (AZD4076), a GalNAc-conjugated anti-miR-103/107 designed for treating NAFLD/NASH, have been launched.^401^ In particular, the phase 1/2a study in T2D patients with NAFLD has been completed (NCT02826525), and a phase 1 study in patients with NASH is now under way (NCT02612662).

#### MicroRNA-22

miR-22 was previously reported as a tumor suppressor to regulate colon and liver cancer.^402^ Elevated miR-22 was observed in the serum of NAFLD patients.^403^ Further studies demonstrated that miR-22 expression was negatively correlated with Fgf21 levels in human or mice with fatty liver, as miR-22 directly targets *FGFR1* 3′ UTR and downregulates *FGF21* transcription through decreasing the recruitment of *PPAR-α* and *PGC-1α*.^404^ In addition, miR-22 was also reported to affect lipogenesis and production of pro-inflammatory cytokines through silencing *SIRT1* transcription,^405^ suggesting that miR-22 inhibition may have therapeutic potential for harnessing NAFLD and obesity by manipulating the metabolic gene-expression landscape. An anti-miR-22 drug candidate, APT-110, was shown to increase insulin sensitivity and effectively reduce hepatic steatosis in mice, suggesting the potential application in NAFLD treatment.^406^ Resalis Therapeutics is currently leading a pre-clinical study to inhibit miR-22 using LNA-based anti-miR-22 (RES-010) for treating NASH/NAFLD.^407^^,^^408^

#### MicroRNA-33

miR-33a was identified as an intronic miRNA located within *SREBP2*, encoding sterol regulatory element binding factor 2 (Srebf2), a transcriptional regulator targeting the expression of cellular cholesterol transporters in cholesterol metabolism.^409^ Studies have shown that the regulation of glucose homeostasis was improved and the development of fibrosis and inflammation was slowed in a hepatic miR-33a deficiency conditional knockout mouse model.^410^ In addition to miR-33a in mice, miR-33b is located in the intron of *SREBP1* in humans,^411^ which is a crucial regulator in hepatic FA synthesis.^412^ Recently, studies have shown that anti-miR-33 treatments, especially anti-miR-33b, ameliorated liver dysfunction and improved the serum and liver lipid profile in Gubra amylin NASH (GAN) diet-induced mice with miR-33b knockin in the intron of *SREBP1*.^413^

#### Krüppel-like factor 11

Serum miR-10b levels in NASH patients have been shown to negatively correlate with the lobular inflammation score.^414^ Its expression was also observed to be significantly lower in the livers of mice with HFD-induced IR.^415^ Mechanistically, miR-10b-5p was shown to upregulate *RTK* (encoding receptor tyrosine kinase) expression through suppressing key transcription factor Krüppel-like factor 11 (Klf11) in interstitial cells of Cajal (ICCs) or pancreatic β cells.^416^^,^^417^ In pre-clinical studies, injection of the miR-10b-5p mimic successfully improved glucose homeostasis and gastrointestinal (GI) motility in mice,^417^ indicating the therapeutic potential of miR-10b-5p mimic in treating metabolic diseases. Led by RosVivo Therapeutics, the development of the miR-10b-5p mimic (RSVI-301) is currently under way for a group of metabolic diseases including NAFLD, T2D, obesity, and GI motility.^418^

## Conclusions and future directions

Thanks to considerable advances in demonstrating underlying links between NASH and various pathological processes including dysregulated lipid metabolism, IR, inflammation, and fibrosis, abundant potential therapeutic targets have been uncovered. However, based on the results from particular clinical trials, Glp-1 analogs were found only to prevent the progression of liver steatosis but not to resolve the pathological changes especially in middle-to-late stage. Relying on advanced chemical modification strategies and a specific liver-targeted delivery system, oligonucleotide drugs are now becoming safe, stable, and selective in therapeutic applications of liver metabolic diseases, including NASH. However, unlike some of the metabolic diseases which are driven by single gene abnormality, NAFLD/NASH is considered a complex syndrome caused by alterations in multiple parameters. Therefore, one key question in this field is whether satisfactory therapeutic efficacy against NASH could be achieved by blocking any single target—in other words, whether multi-target therapies could be applied by combinatory administration of oligonucleotide drugs with conventional drugs. Besides, in addition to the involvement of hepatocytes, other cell types including HSCs and KCs also participate in these processes. However, effective oligonucleotide drug delivery for hepatic non-parenchymal cells remains an obstacle. Therefore, continuous inquiries into more accurate and selective delivery systems are needed. Depending on the results of current clinical and pre-clinical studies on oligonucleotide drugs, the effectiveness and safety of this emerging therapeutic strategy are believed to be open to further improvement, ultimately benefiting NASH patients.
